# Establishment and Comparative Analysis of In Vitro Regeneration Systems for Six Chinese Large-Flowered Chrysanthemum Cultivars

**DOI:** 10.3390/plants15203104

**Published:** 2026-10-10

**Authors:** Zhiyong Wang, Jingjing Zhang, Peiling Li, Lei Liu, Kequn Ma, Li Yang

**Affiliations:** College of Horticulture, Xinyang Agriculture and Forestry University, Xinyang 464000, China; wzhygansu@163.com (Z.W.); 2024190002@xyafu.edu.cn (J.Z.); lxmlpl@163.com (P.L.); 2010190037@xyafu.edu.cn (L.L.); mkq104046@126.com (K.M.)

**Keywords:** Chinese large-flowered chrysanthemum, in vitro regeneration, comprehensive evaluation

## Abstract

*Chrysanthemum morifolium* Ramat. is an ornamental and medicinal plant with important economic value. Conventional propagation restricts its industrial seedling production owing to low efficiency and poor stress adaptability. While tissue culture provides a scalable propagation alternative, systematic comparative analyses of in vitro regeneration across multiple large-flowered Chinese chrysanthemum germplasms remain limited. In this study, six Chinese large-flowered chrysanthemum cultivars were employed to investigate the effects of HgCl_2_ treatment duration on surface sterilization efficiency, the influences of 6-benzylaminopurine (6-BA)/α-naphthalene acetic acid (NAA) concentration ratios on callus induction and adventitious bud differentiation, as well as the regulatory effects of IBA (indole-3-butyric acid) and NAA on rooting. A multi-index evaluation system combining correlation analysis, principal component analysis, and membership function analysis was established to quantitatively assess genotypic differences in regeneration capacity. Significant genotype-dependent tolerance was detected, with optimal sterilization durations of 7 and 9 min. Moderate and low 6-BA/NAA combinations synergistically facilitated explant dedifferentiation and high-quality callus formation, and the combination of 2 mg/L 6-BA and 0.3 mg/L NAA yielded 100% adventitious bud differentiation in ‘Pushuiliubing’, with an average adventitious bud number per explant of 6.09. Exogenous auxin was not essential for rooting, as most cultivars performed well on hormone-free MS medium, with NAA showing higher induction efficacy than IBA. The overall regeneration capacity ranked as ‘Pushuiliubing’ > ‘Zuijin’ > ‘Fushanqiujing’ > ‘Yingfengpiaoling’ > ‘Ziqiangda’ > ‘Tangyutianjiao’. These genotype-specific parameters underpin the industrial propagation and molecular breeding of large-flowered chrysanthemum and inform tissue culture optimization for other *Chrysanthemum* germplasms and closely related ornamental Asteraceae species.

## 1. Introduction

*Chrysanthemum morifolium* Ramat., a perennial herbaceous species of the genus *Chrysanthemum* (Asteraceae) [1,2], ranks among the most commercially dominant ornamental plants worldwide. Endowed with both ornamental and pharmaceutical properties, it has great industrial and economic value and is cultivated extensively across the globe [3]. It is also widely exploited as a medicinal resource in traditional pharmacopeia systems [4,5,6]. Vegetative propagation via stem cuttings and division is routinely employed in commercial chrysanthemum production [7,8]. While these methods enable stable retention of desirable phenotypic traits from donor plants, they are constrained by inherent limitations, including low multiplication efficiency and high vulnerability to biotic and abiotic stressors [9,10], and thus cannot meet the growing demand for large-scale production of high-quality planting materials. Accordingly, there is an urgent need to develop a seedling propagation system featuring minimal environmental disturbance, high reproductive efficiency, and scalable industrial application. Plant tissue culture technology offers a robust solution to these challenges. It also provides a foundational framework for establishing genetic transformation systems, thereby underpinning the biotechnological improvement of horticultural traits in chrysanthemums [11,12,13].

To date, in vitro regeneration systems have been successfully established for numerous cultivated chrysanthemum cultivars worldwide [14,15,16], including ‘Xiaojinhuang’ [17], ‘China Red’ [18], ‘Iwanohakusen’ [19], ‘Jinba’ [20,21], ‘Jinbudiao’ [22],‘Lixiangju’ [23], and ‘Niukou’ (Green) [24], as well as for several wild species, including *Chrysanthemum yoshinaganthum* [25], *Chrysanthemum koraiense* Nakai [26], *Chrysanthemum lavandulifolium* [27], *Chrysanthemum indicum* [28], and *Chrysanthemum vestitum* [29]. Although protocols have been established for a wide range of chrysanthemum cultivars, medium recipes require continuous optimization owing to strong genotypic variation in regeneration capacity. Most existing reports are restricted to individual cultivars, and divergent culture protocols and experimental parameters across independent studies impede the direct cross-comparison of regeneration potential among different germplasms. Consequently, systematic comparative evaluations of multiple large-flowered chrysanthemum germplasms under uniform experimental conditions remain scarce.

Different hormones and plant growth regulators (PGRs), such as 6-benzylaminopurine (6-BA) and α-naphthalene acetic acid (NAA), zeatin (ZT), kinetin (KT), 2,4-dichlorophenoxyacetic acid (2,4-D), thidiazuron (TDZ), and indole-3-butyric acid (IBA), have been employed so far in chrysanthemum tissue culture [30,31]. Stem segments, petals, ray florets, and leaf blades are all commonly used as explants. Among these, leaves from in vitro-grown seedlings are widely used explants for chrysanthemum tissue culture due to easy sampling and stable regeneration performance [32,33]. Overall, the efficiency of chrysanthemum in vitro propagation is not only governed by explant type but is also strongly shaped by the genetic background of cultivars, resulting in marked genotypic variation in regeneration potential [34,35]. Consequently, no universal in vitro regeneration or rapid propagation system is applicable to all chrysanthemum genotypes, and targeted medium optimization is required for individual cultivars. It is imperative to evaluate the regeneration capacity of multiple large-flowered chrysanthemum germplasms under uniform experimental conditions and screen cultivar-specific culture protocols.

In this study, six large-flowered chrysanthemum cultivars—‘Fushanqiujing’, ‘Pushuiliubing’, ‘Tangyutianjiao’, ‘Yingfengpiaoling’, ‘Ziqiangda’, and ‘Zuijin’—were used to establish an in vitro regeneration system via leaf explant tissue culture, with healthy leaves from their aseptic plantlets serving as the source explants. An integrated framework combining correlation analysis, principal component analysis (PCA), and the membership function method was adopted to comprehensively evaluate and rank the in vitro regeneration capacity among the multiple genotypes. The findings not only provide a theoretical basis for the optimization and practical application of in vitro regeneration systems for large-flowered chrysanthemum, but they also lay a robust technical foundation for subsequent investigations into genetic transformation, molecular mechanisms, and biotechnological breeding. These results can provide useful references for optimizing in vitro rapid propagation systems of chrysanthemum and closely related *Chrysanthemum* materials.

## 2. Results

### 2.1. Effect of HgCl_2_ on Explant Sterilization of Chrysanthemum Stem Segments

**Figure 1:**
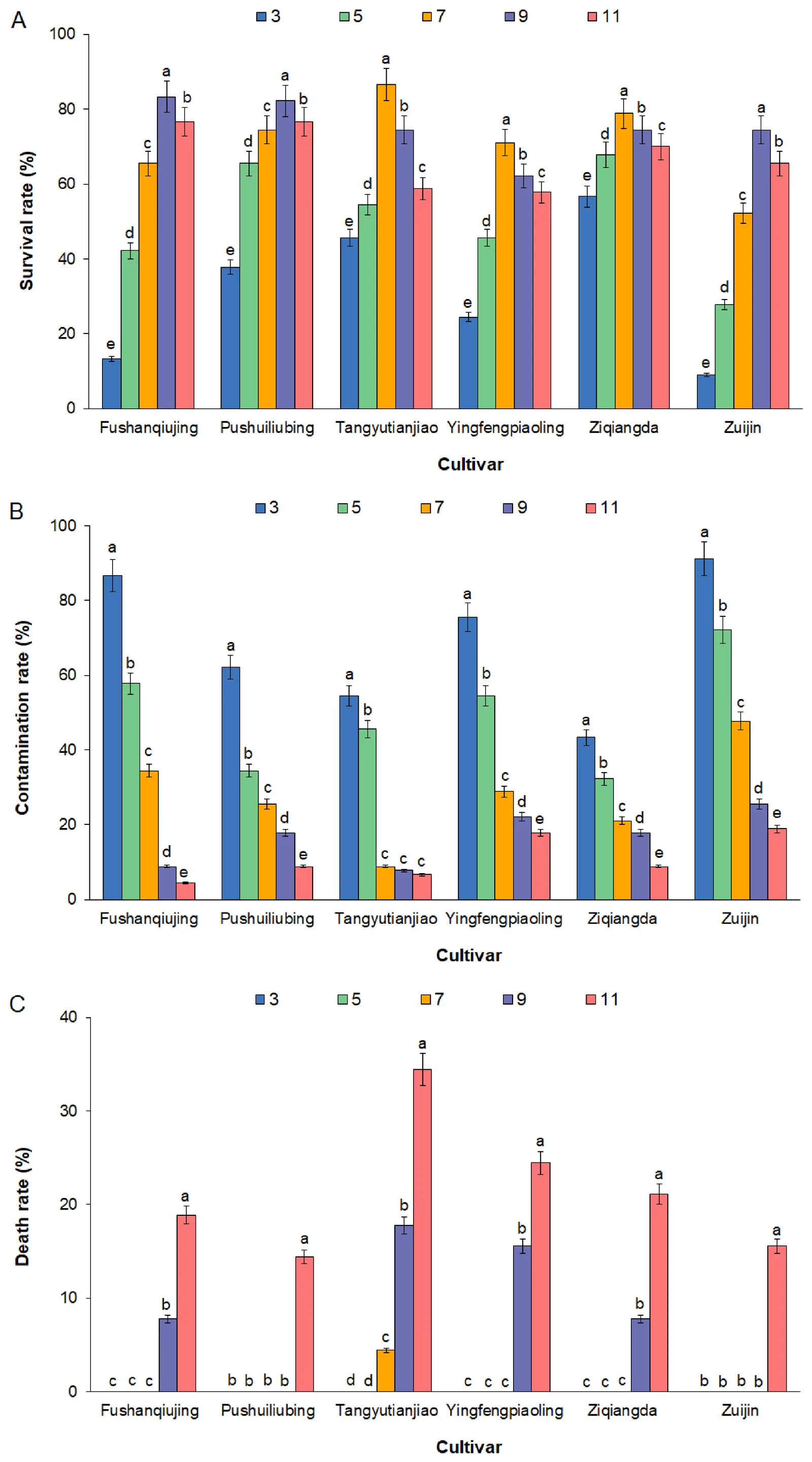
Effects of different HgCl_2_ sterilization durations on the in vitro performance of stem segment explants from six large-flowered chrysanthemum cultivars. (**A**) Survival rate; (**B**) contamination rate; (**C**) death rate. All data are presented as mean ± standard deviation, and error bars represent the standard deviation of the data. Groups with zero values are plotted with minimal bar height for visualization. Numerals 3, 5, 7, 9, and 11 denote HgCl_2_ sterilization duration (min). Different lowercase letters indicate significant differences among treatments within the same cultivar at *p* < 0.05, as determined by Duncan’s multiple range test.

Sterilization using mercuric chloride (HgCl_2_) significantly affected the survival rate, contamination rate, and death rate of stem segment explants from six large-flowered chrysanthemum cultivars. Furthermore, explants of different cultivars displayed distinct genotypic variations in response to the duration of HgCl_2_-based sterilization (Figure 1).

As exposure time to 0.1% HgCl_2_ sterilant was prolonged, the survival rate of stem segment explants of the six chrysanthemum cultivars generally increased first and then decreased (Figure 1A). The 3-min sterilization treatment resulted in low explant survival for all cultivars, with ‘Zuijin’ recording the lowest survival rate of 8.89%. When sterilization duration was extended to 7–9 min, most cultivars reached their maximum survival values, although substantial cultivar-specific differences existed in the optimal sterilization time. For example, ‘Tangyutianjiao’ obtained its highest survival rate (86.67%) after 7 min of sterilization, whereas ‘Fushanqiujing’ achieved its peak survival of 83.33% after 9 min. Further prolonging sterilization duration to 11 min aggravated the phytotoxic effects of HgCl_2_ on stem segment explants, leading to variable reductions in survival rate across all tested chrysanthemum cultivars.

As the 0.1% HgCl_2_ sterilization duration increased from 3 min to 11 min, the contamination rate of stem segment explants continuously declined for all six chrysanthemum cultivars (Figure 1B). The short-duration 3-min sterilization failed to effectively eliminate microbial contamination, which yielded generally high contamination levels in all tested cultivars. Among these cultivars, ‘Zuijin’ showed the highest contamination rate of 91.11%, while ‘Ziqiangda’ had the lowest contamination rate of 43.33%. When sterilization time was extended to 7 min, microbial contamination of explants was markedly suppressed, and contamination rates of all cultivars dropped to 8.89–47.78%. The longest 11-min sterilization treatment delivered superior disinfection efficacy, lowering contamination rates of all cultivars below 20%. In particular, ‘Fushanqiujing’ exhibited the best contamination-control performance, with an extremely low contamination rate of only 4.44%.

The death rate of chrysanthemum stem segment explants varied significantly depending on sterilization duration; HgCl_2_-phytotoxicity-driven mortality mainly occurred in the prolonged 9-min and 11-min treatment groups (Figure 1C). No explant mortality was observed for any of the six cultivars after 3-min and 5-min sterilization treatments. Under the 7-min treatment, only ‘Tangyutianjiao’ presented slight mortality, with a death rate of 4.44%. Once sterilization duration exceeded 9 min, chemical phytotoxicity from HgCl_2_ inflicted irreversible damage on stem segment explants and triggered a sharp rise in explant mortality. Toxic damage became more severe after 11 min of sterilization, which further elevated mortality in all cultivars. Notably, ‘Tangyutianjiao’ showed the highest death rate of 34.44% under the 11-min treatment.

As shown in Table A1, both cultivar and sterilization duration both had highly significant effects on explant survival rate, contamination rate, and death rate (*p* < 0.001). Furthermore, a highly significant interaction effect between the cultivar and sterilization duration was detected for all three parameters. Among the tested factors, sterilization duration was the dominant source of variation for all measured indices, with a substantially larger sum of squares than the cultivar main effect. The interaction between cultivar and sterilization duration was highly significant for all indices. This finding confirms that large-flowered chrysanthemum genotypes exhibit fundamental differences in their responsiveness to 0.1% HgCl_2_ surface sterilization.

Comprehensive evaluation of contamination control efficiency, explant survival status, and death rate revealed that a sterilization duration of 7–9 min was optimal for stem segment disinfection with 0.1% HgCl_2_ solution. Short sterilization times were insufficient to thoroughly remove surface microorganisms, and plants under this treatment consequently maintained high explant contamination rates. In contrast, excessive sterilization exposure induced severe HgCl_2_ phytotoxicity, caused extensive explant death, greatly reduced tissue culture survival efficiency, and ultimately impeded the successful establishment of chrysanthemum stem segment in vitro cultures.

### 2.2. Effect of 6-BA and NAA Concentration Combinations on Callus Induction

Combinations of 6-BA and NAA significantly affected the callus induction rate of stem explants from six large-flowered chrysanthemum cultivars. Meanwhile, these cultivars displayed marked genotypic differences in their responses to such phytohormone combinations (Table 1).

**Figure 2:**
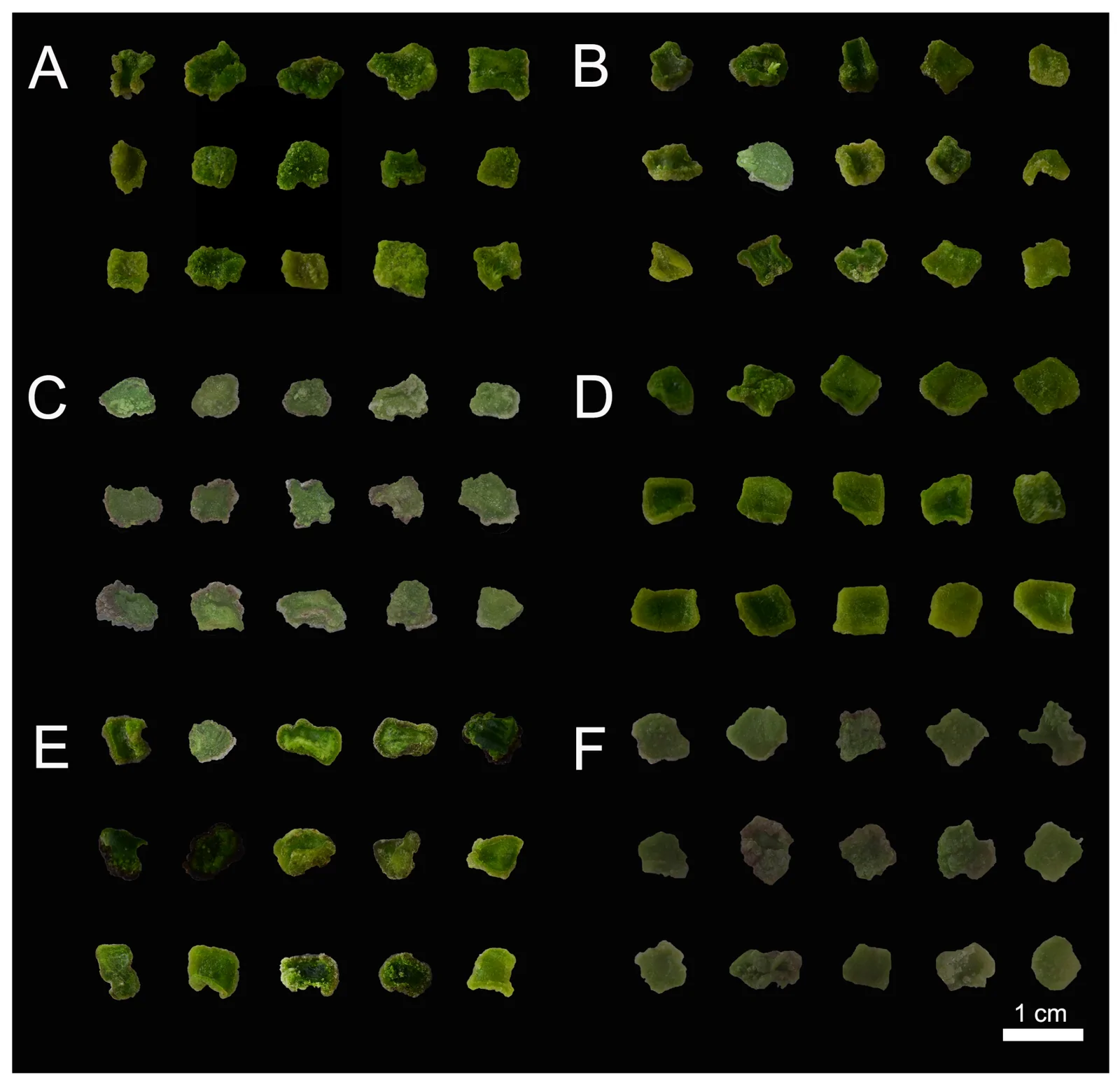
Callus formation of six Chinese large-flowered chrysanthemum cultivars under different 6-BA and NAA combinations. (**A**) Fushanqiujing; (**B**) Pushuiliubing; (**C**) Tangyutianjiao; (**D**) Yingfengpiaoling; (**E**) Ziqiangda; (**F**) Zuijin. For each subgraph, the three rows represent 6-BA concentrations of 1, 2, and 3 mg/L, and the five columns represent NAA concentrations of 0.1, 0.3, 0.5, 0.7, and 0.9 mg/L. Bar = 1 cm.

Under 1 mg/L 6-BA, varying NAA concentrations modified callus induction of leaf disc explants across the six chrysanthemum cultivars. Overall, the callus induction rate first increased and then decreased with rising NAA concentration, with evident genotypic variation. At 0.1 mg/L NAA, all cultivars exhibited low callus induction rates; ‘Fushanqiujing’ reached only 52.22%. The resulting calluses were predominantly pale yellow with loose texture (Figure 2A). Marked improvement in callus induction occurred when NAA concentration ranged from 0.3 to 0.7 mg/L. At 0.3 mg/L NAA, ‘Ziqiangda’ and ‘Pushuiliubing’ obtained callus induction rates of 100% and 97.78%, respectively. Abundant bright green, granular, and compact calluses formed along the margins of leaf disc explants (Figure 2B,E). For ‘Yingfengpiaoling’, 0.7 mg/L NAA yielded 100% callus induction accompanied by vigorous callus proliferation (Figure 2D). Further elevation of NAA to 0.9 mg/L produced no significant additional increase in callus induction rate, and several cultivars generated slightly loose and dark-coloured calluses.

At 2 mg/L 6-BA, the callus induction rate of leaf disc explants was further enhanced for most chrysanthemum cultivars, and the optimal NAA concentration range for callus induction was 0.3–0.5 mg/L. This hormone combination produced the best callus induction performance for ‘Fushanqiujing’, ‘Tangyutianjiao’, ‘Yingfengpiaoling’, and ‘Zuijin’. The treatment of 2 mg/L 6-BA plus 0.30 mg/L NAA achieved a 95.00% callus induction rate in ‘Fushanqiujing’. For ‘Tangyutianjiao’ and ‘Zuijin’, the optimal treatment was 2 mg/L 6-BA plus 0.50 mg/L NAA, with induction rates of 91.67% and 98.89%, respectively. The induced calluses had a bright green colour, compact structure, and uniform growth status (Figure 2A,F). Increasing NAA concentration to 0.7–0.9 mg/L slightly reduced the callus induction rate of all tested cultivars.

At 3 mg/L 6-BA, prominent genotypic differences in leaf disc callus induction were observed among chrysanthemum cultivars. Several cultivars still retained strong callus-inducing capacity under this cytokinin concentration. Nevertheless, high NAA concentration (0.90 mg/L) substantially reduced callus induction efficiency for most genotypes. For ‘Fushanqiujing’ and ‘Tangyutianjiao’, the combination of 3 mg/L 6-BA plus 0.70 mg/L NAA maintained callus induction rates above 90%, and the calluses exhibited favourable growth performance (Figure 2A,C). Once NAA concentration exceeded 0.7 mg/L, undesirable phenotypes emerged in most cultivars, including callus browning, suppressed growth, and loose texture.

As shown in Table A2, cultivar, 6-BA, and NAA all exerted highly significant effects on the callus induction rate of leaf discs from the six large-flowered chrysanthemum cultivars (all *p* < 0.001). All two-way interactions and the three-way interaction among these three factors were also highly significant. Notably, the cultivar × 6-BA interaction made the strongest contribution, with the largest sum of squares among all sources of variation, indicating fundamental genotypic differences in responsiveness to 6-BA concentrations. A significant synergistic interaction was observed between 6-BA and NAA, whose concentration combinations co-regulated callus formation. The cultivar × 6-BA × NAA three-way interaction was likewise highly significant, confirming that the optimal 6-BA/NAA concentration ratio is strongly genotype-dependent and that no universal hormone formulation is suitable for all tested cultivars.

Collectively, appropriate 6-BA/NAA combinations synergistically promote callus initiation and proliferation in chrysanthemum leaf disc explants. Moderate-to-low phytohormone concentrations favour the formation of high-quality callus with compact morphology and vigorous growth. Conversely, excessively high hormone concentrations induce phytotoxicity, leading to callus browning, loose texture, and growth suppression. Accordingly, the optimal callus induction medium for leaf discs differed substantially among cultivars: 2.0 mg/L 6-BA plus 0.3 mg/L NAA for ‘Fushanqiujing’; 1.0 mg/L 6-BA plus 0.3 mg/L NAA for ‘Pushuiliubing’ and ‘Ziqiangda’; 2.0 mg/L 6-BA plus 0.5 mg/L NAA for ‘Tangyutianjiao’ and ‘Zuijin’; and 1.0 mg/L 6-BA plus 0.7 mg/L NAA for ‘Yingfengpiaoling’.

### 2.3. Effect of Combined 6-BA and NAA Concentration on Adventitious Bud Differentiation

Concentrations of 6-BA and NAA significantly modulated adventitious bud differentiation from leaf explants of six large-flowered chrysanthemum cultivars. Distinct genotypes exhibited pronounced differences in their responses to exogenous phytohormones (Table 2 and Table 3). Moderate-to-low 6-BA combined with appropriate NAA synergistically promoted adventitious bud differentiation. In contrast, excessively high hormone concentrations exerted inhibitory effects and lowered differentiation efficiency.

**Figure 3:**
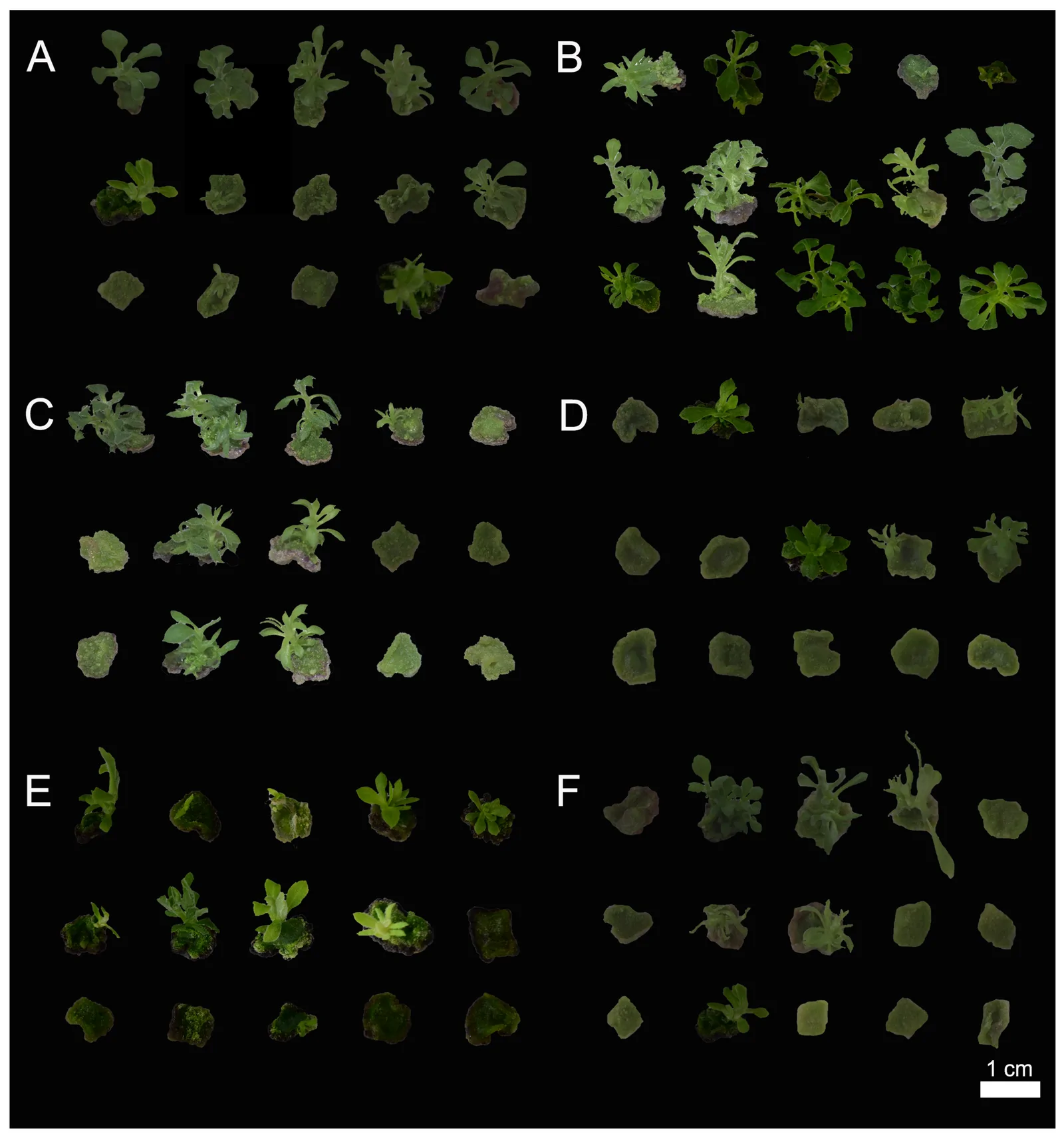
Adventitious bud differentiation of six Chinese large-flowered chrysanthemum cultivars under different 6-BA and NAA combinations. (**A**) Fushanqiujing; (**B**) Pushuiliubing; (**C**) Tangyutianjiao; (**D**) Yingfengpiaoling; (**E**) Ziqiangda; (**F**) Zuijin. Within each subpanel, rows 1–3 correspond to 6-BA concentrations of 1, 2, and 3 mg/L, and columns 1–5 correspond to NAA concentrations of 0.1, 0.3, 0.5, 0.7, and 0.9 mg/L. Bar = 1 cm.

Under 1 mg/L 6-BA, the adventitious bud differentiation rate generally rose first and then declined with increasing NAA concentration across cultivars, although the NAA concentration corresponding to the differentiation peak varied considerably among genotypes. At 0.1 mg/L NAA, all tested cultivars displayed low differentiation rates; no adventitious buds were regenerated in ‘Yingfengpiaoling’ and ‘Zuijin’ (Figure 3D,F). At 0.3 mg/L NAA, ‘Tangyutianjiao’ and ‘Zuijin’ reached their differentiation peaks under this 6-BA level, with adventitious bud differentiation rates of 61.66% and 66.67% and adventitious bud number per explant of 2.52 and 2.72, respectively. The differentiation rate of ‘Pushuiliubing’ also increased to 71.11%, with an adventitious bud number per explant of 0.73. Numerous adventitious buds with favourable growth status were obtained under this hormone combination (Figure 3B,C,F). Further elevation of NAA to 0.7–0.9 mg/L resulted in a continuous increase in the differentiation rate of ‘Yingfengpiaoling’, which peaked at 47.22% with an adventitious bud number per explant of 1.92 at 0.9 mg/L NAA. By comparison, cultivars including ‘Tangyutianjiao’ and ‘Zuijin’ showed varying degrees of reduction in differentiation rate, accompanied by decreased adventitious bud production.

When 6-BA was increased to 2 mg/L, the adventitious bud differentiation potential of most cultivars was further enhanced, and the suitable NAA concentration range was 0.3–0.5 mg/L. ‘Pushuiliubing’ showed the best response to this hormone combination. When treated with 2 mg/L 6-BA plus 0.3 mg/L NAA, it achieved a 100% adventitious bud differentiation rate and the maximum adventitious bud number per explant (6.09) across all treatments, producing robust adventitious buds (Figure 3B). ‘Ziqiangda’ also reached its peak differentiation rate of 37.22% and an adventitious bud number per explant of 0.77 under this combination. ‘Fushanqiujing’, ‘Tangyutianjiao’, and ‘Zuijin’ also exhibited a satisfactory differentiation performance. ‘Fushanqiujing’ attained its peak differentiation rate of 26.11% and an adventitious bud number per explant of 0.59 at 0.9 mg/L NAA. As NAA concentration increased to 0.7–0.9 mg/L, differentiation rates of all cultivars decreased slightly, and the inhibitory effect of high auxin became apparent.

At 3 mg/L 6-BA, genotypic differences in differentiation capacity were further amplified. High cytokinin concentrations imposed substantial inhibitory effects on most cultivars. ‘Yingfengpiaoling’, ‘Ziqiangda’, and ‘Zuijin’ were sensitive to high levels of 6-BA, and few adventitious buds were induced under most treatments. Leaf explants displayed browning, growth arrest, and loose texture, which impeded adventitious bud formation. A small number of cultivars maintained strong differentiation tolerance. Under 3 mg/L 6-BA plus 0.5 mg/L NAA, ‘Pushuiliubing’ retained high differentiation efficiency, with a differentiation rate of 87.23% and an adventitious bud number per explant of 2.72. ‘Tangyutianjiao’ yielded a 41.11% differentiation rate and an adventitious bud number per explant of 1.27 at 0.3 mg/L NAA. ‘Fushanqiujing’ reached a differentiation rate of 18.33% and an adventitious bud number per explant of 0.57 at 0.7 mg/L NAA. Once NAA exceeded 0.7 mg/L, the differentiation efficiency of all tested cultivars decreased significantly, and hormone-derived inhibition was aggravated.

As shown in Table A3, cultivar, 6-BA, and NAA all had highly significant effects on both the adventitious bud differentiation rate and adventitious bud number per explant across the six large-flowered chrysanthemum cultivars (all *p* < 0.001). All two-way and three-way interactions among the three factors also reached highly significant levels. Notably, the cultivar × NAA interaction exerted the strongest influence on differentiation rate, with a sum of squares substantially greater than the main effect of 6-BA, revealing fundamental genotypic differences in responsiveness to auxin concentrations. The cultivar × 6-BA interaction was likewise highly significant, demonstrating that the regulatory role of cytokinin in adventitious bud differentiation is strongly dependent on the cultivar’s genetic background. A significant synergistic interaction was also detected between 6-BA and NAA, whose concentration combinations co-regulated the initiation and development of adventitious buds. The three-way cultivar × 6-BA × NAA interaction was similarly highly significant, confirming that the optimal 6-BA/NAA ratio for adventitious bud differentiation is strongly genotype-dependent.

Based on both the adventitious bud differentiation rate and adventitious bud number per explant, the optimal hormone combinations for adventitious bud differentiation differed markedly among the six large-flowered chrysanthemum cultivars, and no universal medium formula was suitable for all genotypes. ‘Pushuiliubing’ possessed the strongest in vitro differentiation potential, with the optimal medium consisting of MS (Murashige and Skoog) supplemented with 2 mg/L 6-BA and 0.3 mg/L NAA (100% differentiation rate; adventitious bud number per explant 6.09). ‘Zuijin’ and ‘Tangyutianjiao’ preferred relatively low hormone levels, and their optimal medium was MS + 1 mg/L 6-BA + 0.3 mg/L NAA. ‘Yingfengpiaoling’ adapted to a higher auxin proportion, and its optimum combination was MS + 1 mg/L 6-BA + 0.9 mg/L NAA. ‘Ziqiangda’ and ‘Fushanqiujing’ exhibited generally low differentiation efficiency. Their optimal combinations were MS + 2 mg/L 6-BA + 0.3 mg/L NAA and MS + 2 mg/L 6-BA + 0.9 mg/L NAA, respectively.

### 2.4. Effect of IBA or NAA Concentrations on Rooting of Adventitious Shoots

Both IBA and NAA exerted significant regulatory effects on the rooting rate of tissue-cultured plantlets derived from six Chinese large-flowered chrysanthemum cultivars. Distinct genotypes displayed markedly different responses to auxin types and concentrations (Table 4). Under the hormone-free MS control medium, all cultivars generally maintained relatively high rooting rates. Among them, ‘Yingfengpiaoling’ had the highest rooting rate (100.00%), while ‘Pushuiliubing’ had the lowest (80.00%). For single IBA treatment, low concentrations facilitated the rooting of partial chrysanthemum cultivars, whereas high concentrations exerted significant inhibitory effects and induced root phytotoxicity. At the IBA concentration of 0.1 mg/L, the rooting rate of ‘Pushuiliubing’ reached a maximum of 97.78%, while that of ‘Ziqiangda’ dropped to 0. When the IBA concentration increased to 0.3 mg/L or higher, most cultivars completely lost their rooting ability. Only ‘Yingfengpiaoling’ retained rooting capacity under IBA treatments of 0.5–0.7 mg/L. For single NAA application, the rooting rate of most cultivars decreased gradually with the increase in NAA concentration, with obvious genotypic differences in auxin tolerance. ‘Ziqiangda’ sustained high rooting rates of 98.89% and 95.56% under 0.5 mg/L and 0.7 mg/L NAA treatments, respectively. In contrast, rooting was entirely inhibited in ‘Yingfengpiaoling’ and ‘Zuijin’ under the same treatments. Additionally, the rooting rate of ‘Tangyutianjiao’ decreased significantly under elevated NAA concentrations, demonstrating its high sensitivity to high-level NAA. Notably, changes in auxin types and concentrations also caused synchronous variations in mean root number, mean root length, and death rate of tissue-cultured plantlets, further verifying the combined regulatory effect of auxin stress on the rooting performance and growth status of chrysanthemum plantlets.

**Figure 4:**
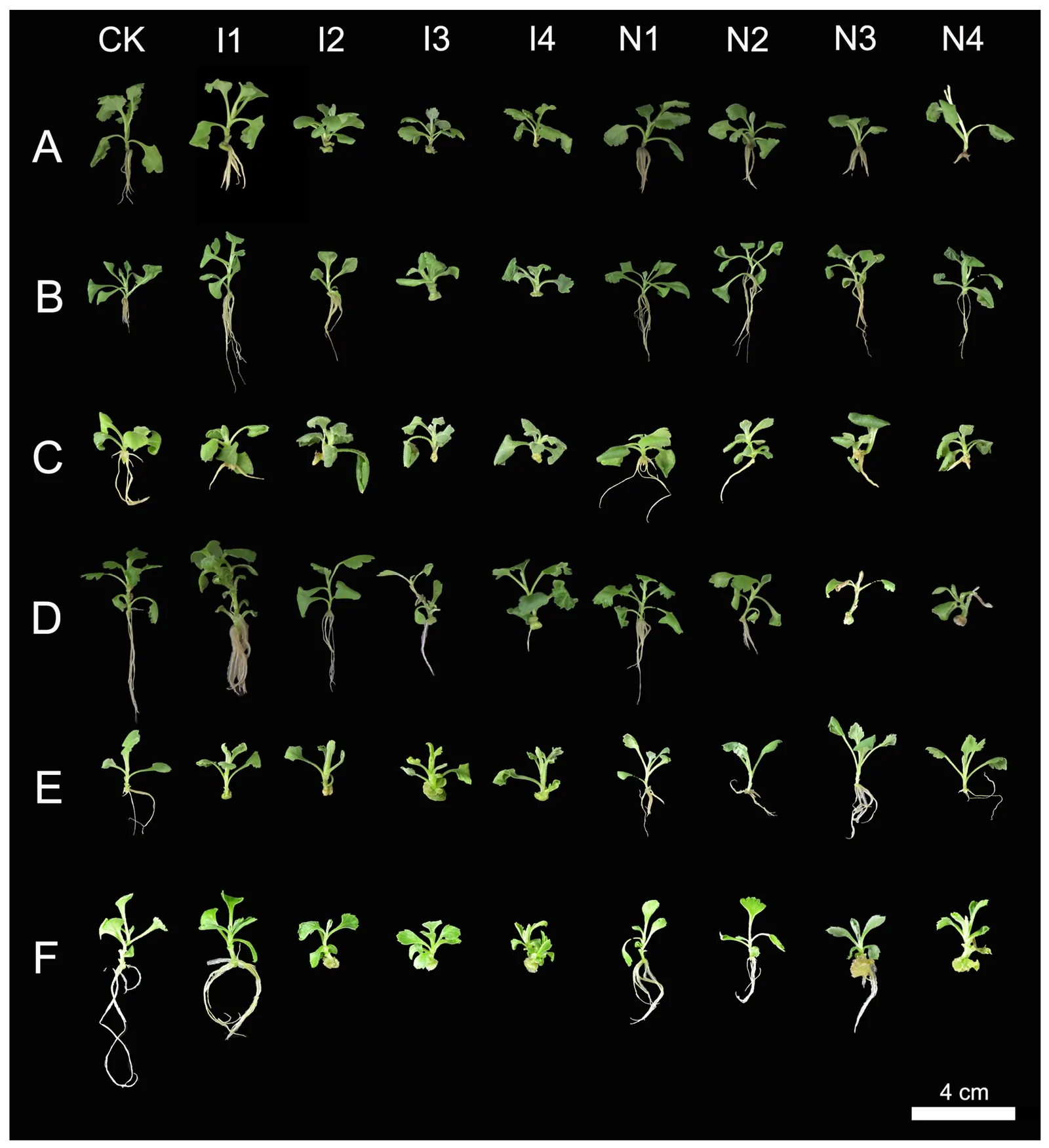
Rooting performance of six Chinese large-flowered chrysanthemum cultivars under different auxin treatments. (**A**) Fushanqiujing; (**B**) Pushuiliubing; (**C**) Tangyutianjiao; (**D**) Yingfengpiaoling; (**E**) Ziqiangda; (**F**) Zuijin. Treatment abbreviations: CK, hormone-free MS medium; I1–I4, IBA treatments at 0.1, 0.3, 0.5, and 0.7 mg/L; N1–N4, NAA treatments at 0.1, 0.3, 0.5, and 0.7 mg/L. Bar = 4 cm.

IBA and NAA exhibited consistent regulatory effects on the adventitious root number and rooting rate, both of which followed a general trend of low-concentration promotion and high-concentration inhibition, with substantial genotypic differences among cultivars (Table 4). Under the hormone-free control condition, ‘Yingfengpiaoling’ produced the highest mean number of adventitious roots (7.95), while ‘Pushuiliubing’ showed the lowest value (4.69). For single IBA treatments, 0.1 mg/L IBA significantly promoted adventitious root formation in ‘Pushuiliubing’, with the mean number of adventitious roots reaching 10.53, which was the maximum value across all treatments. Plantlets of this cultivar also presented vigorous growth status (Figure 4B). In comparison, the remaining cultivars exhibited significantly lower adventitious root numbers than the control group, and no adventitious roots were generated in ‘Ziqiangda’ (Figure 4E). With the increase in IBA concentration, the adventitious root number of all cultivars decreased continuously. Four cultivars, namely ‘Fushanqiujing’, ‘Tangyutianjiao’, ‘Ziqiangda’, and ‘Zuijin’, completely failed to form adventitious roots at 0.3 mg/L IBA. When the IBA concentration was further elevated to 0.5–0.7 mg/L, only ‘Yingfengpiaoling’ retained a small number of adventitious roots (Figure 4D). For single NAA treatments, low NAA concentrations moderately promoted adventitious root formation in ‘Tangyutianjiao’ and ‘Ziqiangda’, whereas inhibitory effects were predominant in the other tested cultivars. Increasing NAA concentration gradually reduced the adventitious root number in most cultivars. Notably, ‘Ziqiangda’ maintained stable adventitious root numbers across the NAA concentration range of 0.1–0.7 mg/L, indicating its exceptionally strong tolerance to exogenous NAA stress.

Adventitious root elongation was also significantly modulated by IBA and NAA. The variation in mean root length was generally consistent with those of rooting rate and mean root number, displaying a pattern of promotion at low concentrations and inhibition at high concentrations, accompanied by prominent genotypic differences (Table 4). Under the hormone-free control, ‘Zuijin’ had the maximum mean root length (48.97 mm), whereas the minimum value was recorded in ‘Ziqiangda’ (21.19 mm). Under single IBA treatments, 0.1 mg/L IBA significantly stimulated root elongation in ‘Pushuiliubing’, yielding a mean root length of 57.64 mm, the highest value observed across all treatments. In contrast, root elongation was markedly suppressed in most other cultivars, and no adventitious roots were formed in ‘Ziqiangda’. The inhibitory effect became more severe as IBA concentration increased. At 0.3 mg/L IBA, mean root length decreased to zero in four cultivars: ‘Fushanqiujing’, ‘Tangyutianjiao’, ‘Ziqiangda’, and ‘Zuijin’. At 0.7 mg/L IBA, only ‘Yingfengpiaoling’ maintained extremely short root systems. For single NAA treatments, 0.1 mg/L NAA significantly promoted root elongation in ‘Tangyutianjiao’. The mean root length of ‘Ziqiangda’ increased slightly with rising NAA concentration, reflecting its strong NAA tolerance. For the remaining cultivars, mean root length declined continuously with increasing NAA concentration, and pronounced inhibitory effects occurred at high auxin concentrations.

As shown in Table A4, cultivar, IBA, and NAA all exerted highly significant main effects on three rooting parameters (rooting rate, mean root number, and mean root length) in in vitro plantlets of the six large-flowered chrysanthemum cultivars (all *p* < 0.001). The two-way interactions of cultivar × IBA and cultivar × NAA reached highly significant levels for all three indices (*p* < 0.001), indicating fundamental genotypic differences in the responsiveness of large-flowered chrysanthemum to IBA and NAA. The regulatory effects of auxins on adventitious root formation, root number accumulation, and root elongation are therefore strongly dependent on the cultivar’s genetic background, and prominent divergence exists in optimal auxin type and concentration among different genotypes. Notably, since only single auxin treatments (IBA or NAA alone) were employed in this experiment with no combined IBA + NAA treatment groups, the sum of squares for both the IBA × NAA two-way interaction and the cultivar × IBA × NAA three-way interaction were zero, exhibiting no statistical significance. This result is fully consistent with the expectation derived from the experimental design.

Comprehensive analysis of rooting rate, mean root number, and mean root length confirmed significant genotypic differences in the rooting capacity and auxin response patterns among the six Chinese large-flowered chrysanthemum cultivars. Both IBA and NAA exhibited typical dose-dependent effects, with low concentrations consistently promoting root growth, and high concentrations consistently inhibiting it. ‘Pushuiliubing’ achieved the optimal comprehensive rooting performance under 0.1 mg/L IBA treatment, with all three rooting indices significantly higher than those of the hormone-free control group, indicating that low-concentration IBA is suitable for rooting induction of this cultivar. ‘Ziqiangda’ was highly sensitive to IBA stress but showed strong tolerance to NAA, maintaining stable and favourable rooting performance across the NAA concentration range of 0.1–0.7 mg/L. ‘Yingfengpiaoling’, ‘Fushanqiujing’, and ‘Zuijin’ exhibited the best rooting performance under the hormone-free condition without exogenous auxin application. By comparison, ‘Tangyutianjiao’ showed superior rooting ability under low-concentration NAA treatment and was highly susceptible to growth inhibition caused by high levels of exogenous auxins.

### 2.5. Establishment of Six Chinese Large-Flowered Chrysanthemum Cultivars

**Figure 5:**
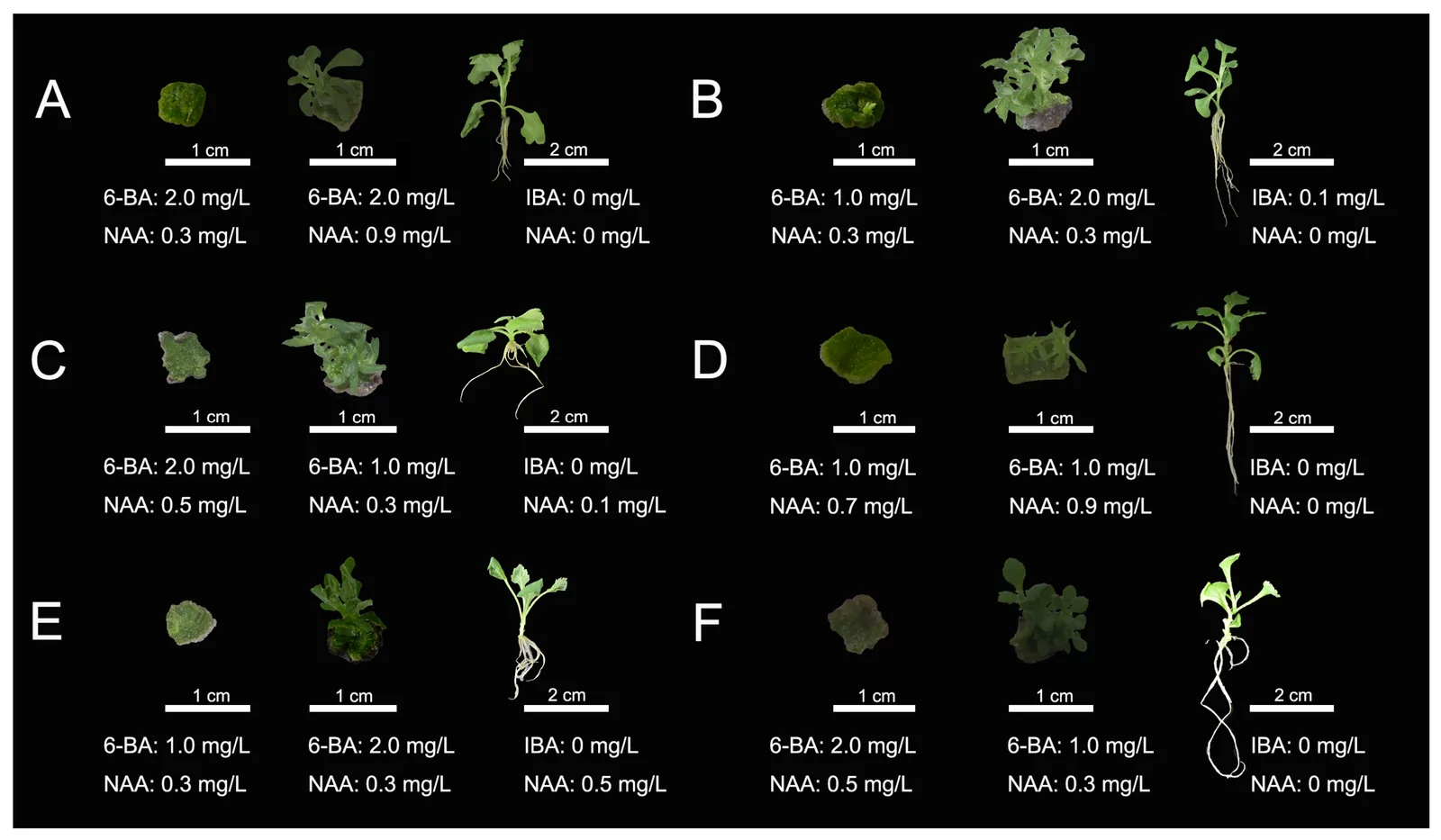
Regeneration system for six large-flowered chrysanthemum cultivars. (**A**) Fushanqiujing; (**B**) Pushuiliubing; (**C**) Tangyutianjiao; (**D**) Yingfengpiaoling; (**E**) Ziqiangda; (**F**) Zuijin.

Based on experiments on stem segment explant sterilization, leaf disc callus induction, adventitious bud differentiation, and rooting stages, this study established a preliminary in vitro regeneration system for six large-flowered chrysanthemum cultivars. The overall workflow of in vitro leaf regeneration was consistent across all six large-flowered chrysanthemum cultivars. Stem segment explants were surface-sterilized with 0.1% HgCl_2_ and cultured for 30 d to generate aseptic plantlets. Healthy leaves were excised into 0.5 cm × 0.5 cm leaf discs, which were first cultured on callus induction medium for 3 weeks, transferred to adventitious bud differentiation medium for another 3 weeks, and finally subcultured on rooting medium for 15 d. All cultivars produced fully regenerated plantlets with vigorous growth and well-developed root systems. The optimal culture parameters for each cultivar are detailed below:

‘Fushanqiujing’: Optimal 0.1% HgCl_2_ sterilization duration for stem segments: 9 min; callus induction medium: MS basal medium + 2.0 mg/L 6-BA + 0.3 mg/L NAA; adventitious bud differentiation medium: MS basal medium + 2.0 mg/L 6-BA + 0.9 mg/L NAA; rooting condition: hormone-free MS basal medium (Figure 5A).

‘Pushuiliubing’: Optimal 0.1% HgCl_2_ sterilization duration for stem segments: 9 min; callus induction medium: MS basal medium + 1.0 mg/L 6-BA + 0.3 mg/L NAA; adventitious bud differentiation medium: MS basal medium + 2.0 mg/L 6-BA + 0.3 mg/L NAA; rooting medium: MS basal medium supplemented with 0.1 mg/L IBA (Figure 5B).

‘Tangyutianjiao’: Optimal 0.1% HgCl_2_ sterilization duration for stem segments: 7 min; callus induction medium: MS basal medium + 2.0 mg/L 6-BA + 0.5 mg/L NAA; adventitious bud differentiation medium: MS basal medium + 1.0 mg/L 6-BA + 0.3 mg/L NAA; rooting medium: MS basal medium supplemented with 0.1 mg/L NAA (Figure 5C).

‘Yingfengpiaoling’: Optimal 0.1% HgCl_2_ sterilization duration for stem segments: 7 min; callus induction medium: MS basal medium + 1.0 mg/L 6-BA + 0.7 mg/L NAA; adventitious bud differentiation medium: MS basal medium + 1.0 mg/L 6-BA + 0.9 mg/L NAA; rooting condition: hormone-free MS basal medium (Figure 5D).

‘Ziqiangda’: Optimal 0.1% HgCl_2_ sterilization duration for stem segments: 7 min; callus induction medium: MS basal medium + 1.0 mg/L 6-BA + 0.3 mg/L NAA; adventitious bud differentiation medium: MS basal medium + 2.0 mg/L 6-BA + 0.3 mg/L NAA; rooting medium: MS basal medium supplemented with 0.5 mg/L NAA (Figure 5E).

‘Zuijin’: Optimal 0.1% HgCl_2_ sterilization duration for stem segments: 9 min; callus induction medium: MS basal medium + 2.0 mg/L 6-BA + 0.5 mg/L NAA; adventitious bud differentiation medium: MS basal medium + 1.0 mg/L 6-BA + 0.3 mg/L NAA; rooting condition: hormone-free MS basal medium (Figure 5F).

### 2.6. Comprehensive Evaluation of In Vitro Regeneration Capacity of Six Chinese Large-Flowered Chrysanthemum Cultivars

This comprehensive evaluation was designed to assess the overall in vitro tissue culture suitability of each cultivar (genotype), rather than focusing on traits derived from a single explant type. The stem segment sterilization experiment reflected genotypic differences in tolerance to mercuric chloride disinfection and contamination resistance, which constitutes a key cultivar attribute for routine tissue culture operations. Subsequent regeneration assays employed leaf explants to characterize genotype-dependent regeneration capacity. Accordingly, both sterilization-associated traits and leaf regeneration performance were integrated into the multi-index evaluation system to comprehensively compare the overall tissue culture performance of the six tested cultivars.

**Figure 6:**
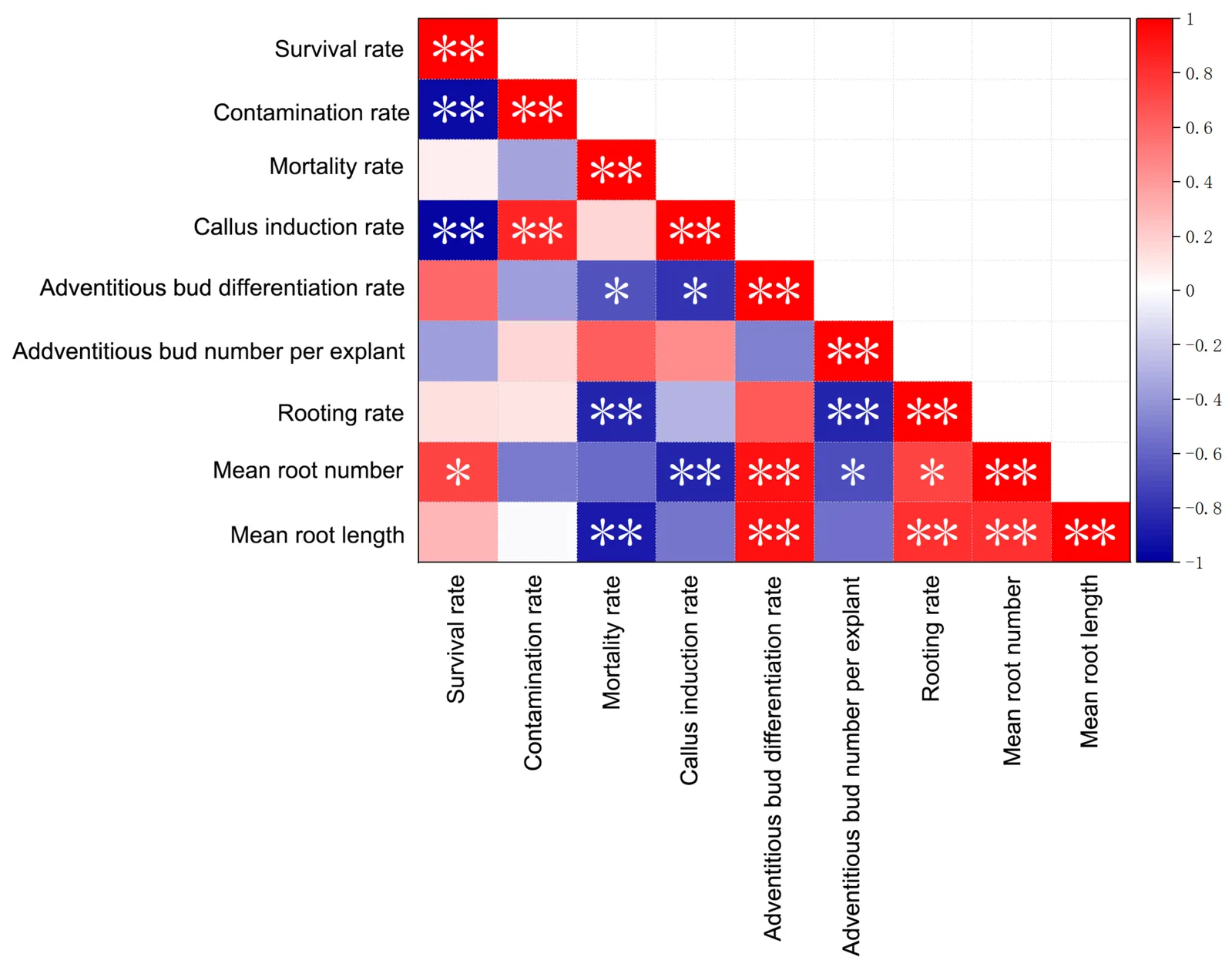
Pearson correlation analysis of nine in vitro regeneration indices for six Chinese large-flowered chrysanthemum cultivars. Note: * and ** indicate significant correlations at *p* < 0.05 and *p* < 0.01, respectively.

Pearson correlation analysis was performed for the nine indices of six Chinese large-flowered chrysanthemum cultivars, and the corresponding correlation results are shown in Figure 6. The survival rate exhibited a highly significant negative correlation with contamination rate and callus induction rate, as well as a significant positive correlation with mean root number. The contamination rate was highly positively correlated with callus induction rate. In addition, the death rate was significantly negatively correlated with adventitious bud differentiation rate and showed highly significant negative correlations with rooting rate and mean root length. The callus induction rate was significantly negatively correlated with adventitious bud differentiation rate and highly negatively correlated with mean root number. Adventitious bud differentiation rate was highly significantly positively correlated with both mean root number and mean root length. The adventitious bud number per explant was highly negatively correlated with rooting rate and significantly negatively correlated with mean root number. Moreover, the rooting rate was significantly positively correlated with mean root number and highly significantly positively correlated with mean root length. A highly significant positive correlation was also observed between mean root number and mean root length.

PCA was conducted based on nine physiological indices. Three principal components (PCs) were extracted according to the eigenvalue-greater-than-one criterion (Table 5). Accordingly, the original nine individual indices were transformed into three new and mutually independent comprehensive factors. PC1, PC2, and PC3 accounted for 45.181%, 31.295%, and 14.503% of the total variance, respectively, with a cumulative variance contribution rate of 90.979%.

Based on the PCA results of nine individual indices, membership function values for the three comprehensive indicators were calculated using Formula (1) (Table 6). The weights of the three principal components, calculated via Formula (2), were 0.497, 0.344, and 0.159. Subsequently, the comprehensive evaluation value (i.e., the *D*-value), which was used to quantify the in vitro regeneration capacity of the six Chinese large-flowered chrysanthemum cultivars, was determined using Formula (3). In vitro regeneration capacity was ranked in descending order according to the *D*-values (the comprehensive evaluation value) as follows: ‘Pushuiliubing’ > ‘Zuijin’ > ‘Fushanqiujing’ > ‘Yingfengpiaoling’ > ‘Ziqiangda’ > ‘Tangyutianjiao’. Among the tested cultivars, ‘Pushuiliubing’ presented the highest *D*-value of 0.853, reflecting its superior in vitro regeneration capacity. In comparison, ‘Tangyutianjiao’ had the lowest *D*-value of 0.197, corresponding to the weakest regeneration performance.

## 3. Discussion

Explant surface sterilization is fundamental for reliable in vitro plant propagation, as residual epiphytic microorganisms readily trigger contamination and culture failure. Owing to its strong oxidizing and bactericidal activity, 0.1% mercuric chloride (HgCl_2_) has been widely adopted for explant disinfection in plant tissue culture [36,37,38]. Consistent with conventional tissue culture principles, HgCl_2_ sterilization exerted a typical dual effect on chrysanthemum leaf explants along with prolonged treatment duration: microbial contamination was gradually eliminated, whereas cumulative phytotoxicity was aggravated, leading to a fluctuating survival phenotype with an initial increase and subsequent sharp decline. Sterilization for 3 min was inadequate for thorough decontamination, with the cultivar ‘Zuijin’ recording a contamination rate above 90% that completely blocked subsequent culture progression. The 7–9 min treatment window achieved an optimal balance between disinfection efficacy and tissue physiological activity, efficiently removing surface contaminants, alleviating oxidative damage, and maximizing explant survival across all six Chinese large-flowered chrysanthemum cultivars. In contrast, excessive sterilization for 11 min caused irreversible tissue injury and substantially increased explant mortality, despite effective contamination control. Notably, distinct genotypic variation in HgCl_2_ tolerance was observed among cultivars, resulting in differentiated optimal treatment durations: 7 min for ‘Tangyutianjiao’, ‘Yingfengpiaoling’, and ‘Ziqiangda’ and 9 min for ‘Fushanqiujing’, ‘Pushuiliubing’, and ‘Zuijin’. These results verify that universal sterilization protocols are poorly adaptable for diverse chrysanthemum germplasms, and genotype-dependent parameter optimization is indispensable for constructing stable and reproducible in vitro regeneration systems. Moreover, the optimal 7–9 min HgCl_2_ exposure range determined in this study is consistent with the documented effective interval of 4–10 min for chrysanthemum and other Asteraceae species [39,40,41], demonstrating conserved physiological stress responses to HgCl_2_ within Asteraceae plants. Collectively, the cultivar-specific sterilization parameters established in this work validate traditional disinfection strategies and provide refined and targeted technical references for high-efficiency tissue culture, large-scale seedling propagation, and germplasm utilization of large-flowered chrysanthemum. However, HgCl_2_ is inherently a highly toxic heavy metal reagent with notable limitations in practical application. It poses health risks linked to occupational exposure during handling, and overexposure readily induces explant browning and cellular damage. Furthermore, mercury-containing laboratory waste has high environmental persistence and requires strict disposal protocols. These limitations have become increasingly prominent as laboratory environmental protection regulations continue to tighten. To address these concerns, future studies could systematically optimize the treatment parameters of low-toxicity sterilants (e.g., sodium hypochlorite and hydrogen peroxide) based on the structural characteristics of chrysanthemum explants, or explore the application potential of eco-friendly sterilization agents such as silver nanoparticles. Such efforts would support the gradual development of a surface sterilization system for chrysanthemum tissue culture explants that balances disinfection efficacy and ecological safety.

The 6-BA/NAA cytokinin–auxin combination is the standard regimen for chrysanthemum callus induction, with their concentration ratio critically regulating explant dedifferentiation and callus quality [42,43,44]. Suboptimal hormone dosages strongly impair regeneration: low concentrations cannot initiate cell dedifferentiation, while excessive doses induce phytotoxicity characterized by tissue browning, loose callus structure, and arrested growth [45,46]. In this study, callus induction exhibited a unimodal response to increasing NAA levels under 1 mg/L 6-BA, with the 0.3–0.7 mg/L NAA range generating compact, green, and vigorously proliferating calluses. Increasing 6-BA to 2 mg/L further improved induction efficiency in most cultivars, including ‘Fushanqiujing’, ‘Tangyutianjiao’, ‘Yingfengpiaoling’, and ‘Zuijin’, and narrowed the optimal NAA range to 0.3–0.5 mg/L, indicating that elevated cytokinin availability lowers the auxin requirement for effective dedifferentiation. Although 3 mg/L 6-BA still sustained moderate callus formation in certain genotypes, NAA concentrations above 0.7 mg/L triggered severe browning and growth inhibition, confirming that excess auxin disrupts cellular homeostasis and suppresses regeneration. These findings are in agreement with those previously documented for *Quercus palustris*, in which high concentrations of NAA were also shown to exert an inhibitory effect on callus induction [47], and moderate-to-low 6-BA/NAA ratios produced synergistic effects to facilitate high-quality callus formation. The divergent responses to PGRs across cultivars could potentially be attributed to genotypic variation in endogenous hormone metabolism and hormone signal sensitivity, although these physiological parameters were not quantified in the present study. Such genetic variability resulted in genotype-specific optimal hormone formulas, demonstrating that no universal PGR protocol is applicable to all large-flowered chrysanthemum cultivars and highlighting the importance of tailored hormone optimization for stable tissue culture systems.

Adventitious bud differentiation represents the core morphogenetic step for establishing stable in vitro chrysanthemum regeneration systems, in which cytokinins primarily trigger bud primordium initiation, while auxins synergistically modulate cell division and tissue morphogenesis. The combinatorial application of 6-BA and NAA has been well documented as a pivotal regulatory module for plant dedifferentiation and adventitious regeneration [48,49,50,51], and this synergistic hormone pattern has been widely validated in multiple chrysanthemum tissue culture studies [24,31,52,53]. Nevertheless, due to distinct genetic backgrounds, chrysanthemum cultivars exhibit highly variable responsiveness to exogenous hormone ratios, leading to genotype-dependent optimal PGR regimens. Previous studies have confirmed substantial inter-cultivar differences in regeneration performance: the cultivar ‘Yingjie Hongfei’ achieved a 70.76% bud induction rate under 2.0 mg/L 6-BA + 0.6 mg/L NAA [32], whereas ‘Jianglingjinju’ only obtained a low regeneration rate (13.33%) and propagation coefficient (0.2) at 1.0 mg/L 6-BA + 0.5 mg/L NAA [54], further demonstrating that genotype dominates in vitro efficiency in *Chrysanthemum morifolium*. Consistent with these findings, our results revealed a cultivar-specific regeneration response to graded 6-BA and NAA combinations. Under 1 mg/L 6-BA, the adventitious bud differentiation rate of most cultivars displayed a unimodal trend with increasing NAA concentration, while the peak-value NAA dosage varied distinctly across genotypes. Increasing 6-BA to 2 mg/L substantially improved the overall regeneration potential and narrowed the optimal NAA range to 0.3–0.5 mg/L. Notably, the cultivar ‘Pushuiliubing’ achieved a maximum 100% differentiation rate and a high adventitious bud number per explant of 6.09 at 2 mg/L 6-BA combined with 0.3 mg/L NAA, representing the most efficient regeneration formula observed in this study. By contrast, elevated cytokinin at 3 mg/L induced obvious phytotoxicity and regeneration inhibition in most cultivars, with only a small number of genotypes retaining moderate differentiation capacity. Based on a comprehensive evaluation of differentiation rate and adventitious bud number per explant, the six Chinese large-flowered chrysanthemum cultivars possessed divergent optimal hormone regimens and variable regeneration competence. ‘Pushuiliubing’ exhibited superior in vitro regeneration performance, while ‘Ziqiangda’ and ‘Fushanqiujing’ showed relatively low differentiation efficiency. Such prominent genotypic divergence is most likely attributed to inherent differences in endogenous hormone homeostasis and exogenous hormone signal sensitivity among chrysanthemum germplasms, further showing that genotype is the decisive factor governing chrysanthemum adventitious bud regeneration capacity.

Auxins act as central regulators of root induction and are indispensable for adventitious root formation during in vitro plant regeneration [33,55]. IBA and NAA are the two most commonly used exogenous auxins in tissue culture; they modulate cell division and elongation to govern adventitious root quantity, root length, and root morphogenesis, whereas excessive auxin doses trigger phytotoxicity and impair normal root development. In the present study, most large-flowered chrysanthemum cultivars retained relatively high rooting efficiency on hormone-free MS medium, demonstrating that endogenous auxins produced by regenerated plantlets can sustain basic adventitious root initiation and development. For sole IBA treatments, only the low concentration (0.1 mg/L) promoted rooting in ‘Pushuiliubing’, yielding the maximum rooting rate, adventitious root number, and average root length among all tested treatments. Root growth was rapidly suppressed as IBA concentration increased. This has been observed in previous work on *Chrysanthemum morifolium* cv. Chuju, where 0.1 mg/L IBA was likewise identified as optimal for rooting induction [55]. When NAA was supplied alone, cultivars displayed more striking genotypic divergence in auxin tolerance. ‘Ziqiangda’ showed robust NAA tolerance and maintained favourable rooting performance across a wide concentration range (0.1–0.7 mg/L). By comparison, ‘Yingfengpiaoling’ and ‘Zuijin’ were highly sensitive to NAA dosage changes, and high NAA concentrations completely blocked rooting. Overall, NAA was more suitable for in vitro rooting of the tested large-flowered chrysanthemum cultivars than IBA, consistent with observations reported for pyrethrum [56]. Collectively, although chrysanthemum cultivars show extensive genotypic variation in optimal rooting regimes, certain genotypes can share compatible rooting medium formulations.

Most current studies concerning the in vitro regeneration of chrysanthemum and other Asteraceae species predominantly focus on system optimization for individual cultivars [41,44,57], whereas systematic comparative evaluations of regeneration performance across multiple genotypes remain limited. Plant in vitro regeneration is a continuous and holistic biological process, in which key phenotypic indicators from sterilization, callus induction, adventitious bud differentiation, and rooting stages are intrinsically interconnected and mutually constrained. Single-indicator evaluation is therefore insufficient to objectively reflect the overall regeneration competence of different germplasms. In contrast, multi-indicator comprehensive assessment enables more scientific and credible quantification of genotype-specific regeneration capacity. Here, PCA was combined with the membership function method to comprehensively evaluate the in vitro regeneration performance of six Chinese large-flowered chrysanthemum cultivars. Nine individual regeneration indicators were simplified into three independent comprehensive components, and the derived comprehensive *D*-values were used to quantify and rank the overall regeneration capacity of the tested cultivars. The final genotypic ranking of in vitro regeneration potential was determined as follows: ‘Pushuiliubing’ > ‘Zuijin’ > ‘Fushanqiujing’ > ‘Yingfengpiaoling’ > ‘Ziqiangda’ > ‘Tangyutianjiao’. This multi-dimensional evaluation strategy effectively avoids the one-sided bias of traditional single-parameter assessment, providing a reliable quantitative framework for screening superior chrysanthemum germplasms and evaluating tissue culture adaptability across diverse genotypes.

In the present study, we successfully obtained in vitro rooted plantlets of six chrysanthemum cultivars and comprehensively evaluated the regeneration capacity of different treatments using membership function analysis. However, acclimatization and transplantation experiments were not conducted in this work, and the transplant survival performance of these regenerated plantlets needs to be explored in further research. Additionally, the present study performed PCA on only six large-flowered chrysanthemum cultivars using nine evaluation indices. The relatively limited number of cultivars restricts the generalizability of the findings. Accordingly, the established multi-index evaluation system and the resulting cultivar regeneration ranking are applicable exclusively to the six tested genotypes. Future studies incorporating a broader panel of chrysanthemum germplasms are warranted to validate the stability and universality of this evaluation framework.

## 4. Materials and Methods

### 4.1. Plant Material, Basal Medium, and Growth Conditions

Six large-flowered *Chrysanthemum morifolium* cultivars, namely ‘Fushanqiujing’, ‘Pushuiliubing’, ‘Tangyutianjiao’, ‘Yingfengpiaoling’, ‘Ziqiangda’, and ‘Zuijin’, were used in this study. All plant materials were maintained and provided by the Experimental Training Base, College of Horticulture, Xinyang Agriculture and Forestry University, Xinyang, Henan Province, China. MS medium [58] was adopted as the basal medium, supplemented with 3% (*w*/*v*) sucrose and 0.7% agar. The pH was adjusted to 5.8 using 1.0 mol/L NaOH prior to autoclaving. 6-BA, NAA, and IBA were purchased from Macklin (Shanghai, China). All explants and regenerated plantlets were cultured in a phytotron at 23 ± 2 °C under a 12 h light/12 h dark photoperiod, with a light intensity of 50 μmol m^−2^ s^−1^.

### 4.2. Sterilization of Explants and Production of Aseptic Seedlings

The sterilization experiment was carried out to obtain contamination-free explants with high survival rate for subsequent in vitro regeneration culture. Young stem segments with axillary buds from healthy mother plants were used as explants. Explants were rinsed under running tap water for 30 min, followed by surface disinfection with 75% ethanol for 30 s. Then, they were treated with 0.1% (*w*/*v*) HgCl_2_ for 3, 5, 7, 9, or 11 min with continuous shaking to achieve adequate surface sterilization. Explants were then rinsed five times with 100 mL sterile water, 3 min for each wash. After rinsing, explants were placed on sterile filter paper to remove surface-adhered water. Both wounded ends were trimmed, and the processed explants were inoculated onto hormone-free MS medium. All cultures were maintained under the same growth conditions (temperature, photoperiod and light intensity) as described in Section 4.1. Each treatment contained three biological replicates with 30 explants per replicate. Survival rate, contamination rate, and death rate were recorded after 14 d of culture. A large number of vigorously growing aseptic seedlings were obtained after 30 d of culture.

### 4.3. Callus Induction from Leaves of Aseptic Seedlings

Fully expanded leaves excised from aseptic chrysanthemum seedlings were used as explants to evaluate the combined effects of 6-BA (1.0, 2.0, 3.0 mg/L) and NAA (0.1, 0.3, 0.5, 0.7, 0.9 mg/L) on callus formation. Leaves were cut into uniform 0.5 cm × 0.5 cm leaf disc explants and inoculated onto culture media with the adaxial side facing upward. The basal medium and general incubation conditions were consistent with those described in Section 4.2. Each treatment included 60 leaf discs, and all experiments were performed in triplicate. Callus induction rate was scored after three weeks of culture.

### 4.4. Adventitious Bud Differentiation

Leaf disc explants with pre-induced callus were subcultured onto MS medium supplemented with varying concentrations of 6-BA and NAA for adventitious bud differentiation. A full factorial experimental design was adopted with three 6-BA concentration levels (1.0, 2.0, and 3.0 mg/L) and five NAA concentration levels (0.1, 0.3, 0.5, 0.7, and 0.9 mg/L), generating a total of 15 hormone treatment combinations. Sixty leaf discs were inoculated per treatment, and the experiment was conducted with three independent biological replicates. Adventitious bud differentiation rate and adventitious bud number per explant were recorded after six weeks of culture.

### 4.5. Adventitious Shoot Rooting

Adventitious shoots of approximately 1.5 cm in height were excised and transferred to MS-based rooting media supplemented with different concentrations of NAA or IBA (0.1, 0.3, 0.5, 0.7 mg/L). Each treatment comprised three replicates with 30 explants per replicate. After 15 d of culture, rooting rate, mean root number, and mean root length of adventitious shoots were determined.

### 4.6. Statistical Analysis

Raw data were compiled in Microsoft Office Excel (Microsoft Corp., Redmond, WA, USA). All statistical analyses were performed using SPSS 27.0 (IBM Corp., Chicago, IL, USA). Two-way factorial ANOVA with cultivar and sterilization duration as fixed factors was applied for sterilization indices. Three-way factorial ANOVAs were used for callus induction/adventitious bud differentiation (cultivar, 6-BA, NAA) and rooting (cultivar, IBA, NAA), all testing main and interaction effects. Results are presented as mean ± standard deviation. Duncan’s multiple range test was used for multiple comparisons at *p* < 0.05. The formulae for calculation are listed below:

$$\mathrm{Survival}\;\mathrm{rate}\;\left(\%\right)=\frac{\mathrm{Number}\;\mathrm{of}\;\mathrm{uncontaminated}\;\mathrm{surviving}\;\mathrm{explants}}{\mathrm{Total}\;\mathrm{number}\;\mathrm{of}\;\mathrm{inoculated}\;\mathrm{explants}}\times 100$$

$$\mathrm{Contamination}\;\mathrm{rate}\;\left(\%\right)=\frac{\mathrm{Number}\;\mathrm{of}\;\mathrm{contaminated}\;\mathrm{explants}}{\mathrm{otal}\;\mathrm{number}\;\mathrm{of}\;\mathrm{inoculated}\;\mathrm{explants}}\times 100$$

$$\mathrm{Death}\;\mathrm{rate}\;\left(\%\right)=\frac{\mathrm{Number}\;\mathrm{of}\;\mathrm{uncontaminated}\;\mathrm{but}\;\mathrm{dead}\;\mathrm{explants}}{\mathrm{Total}\;\mathrm{number}\;\mathrm{of}\;\mathrm{inoculated}\;\mathrm{explants}}\times 100$$

$$\mathrm{Callus}\;\mathrm{induction}\;\mathrm{rate}\;\left(\%\right)=\frac{\mathrm{Number}\;\mathrm{of}\;\mathrm{explants}\;\mathrm{producing}\;\mathrm{callus}}{\mathrm{Total}\;\mathrm{number}\;\mathrm{of}\;\mathrm{uncontaminated}\;\mathrm{explants}\;\mathrm{in}\;\mathrm{the}\;\mathrm{same}\;\mathrm{treatment}}\times 100$$

$$\mathrm{Adventitious}\;\mathrm{bud}\;\mathrm{differentiation}\;\mathrm{rate}\;\left(\%\right)=\frac{\mathrm{Number}\;\mathrm{of}\;\mathrm{explants}\;\mathrm{producing}\;\mathrm{adventitious}\;\mathrm{buds}}{\mathrm{Total}\;\mathrm{number}\;\mathrm{of}\;\mathrm{uncontaminated}\;\mathrm{explants}\;\mathrm{in}\;\mathrm{the}\;\mathrm{same}\;\mathrm{treatment}}\times 100$$

$$\mathrm{Adventitious}\;\mathrm{bud}\;\mathrm{number}\;\mathrm{per}\;\mathrm{explant}=\frac{\mathrm{Total}\;\mathrm{number}\;\mathrm{of}\;\mathrm{regenerated}\;\mathrm{adventitious}\;\mathrm{buds}}{\mathrm{Number}\;\mathrm{of}\;\mathrm{uncontaminated}\;\mathrm{explants}\;\mathrm{in}\;\mathrm{the}\;\mathrm{same}\;\mathrm{treatment}}$$

$$\mathrm{Rooting}\;\mathrm{rate}\;\left(\%\right)=\frac{\mathrm{Number}\;\mathrm{of}\;\mathrm{rooted}\;\mathrm{plantlets}}{\mathrm{Total}\;\mathrm{number}\;\mathrm{of}\;\mathrm{uncontaminated}\;\mathrm{plantlets}\;\mathrm{in}\;\mathrm{the}\;\mathrm{same}\;\mathrm{treatment}}\times 100$$

### 4.7. Comprehensive Evaluation

The membership function method was applied to comprehensively assess the in vitro regeneration capacity of the six large-flowered *C. morifolium* cultivars. Firstly, mean values of nine regeneration-related indices, including survival rate, contamination rate, death rate, callus induction rate, adventitious bud differentiation rate, adventitious bud number per explant, rooting rate, mean root number, and mean root length, were calculated across all hormone treatments for each cultivar. Pearson correlation analysis and PCA were subsequently conducted to obtain eigenvectors of each index and the contribution rate of each principal component. The membership function value for each principal component was calculated according to Formula (1); index weight was calculated via Formula (2); and *D*-value was computed using Formula (3) to quantitatively compare regeneration performance among cultivars.

(1) $$U(X_{i})=(X_{i}-X_{\min})/(X_{\max}-X_{\min})\;\;\;\;\;i=1,\;2,\;\ldots \ldots \;n$$

where $U(X_{i})$ represents the membership function value, $X_{i}$ is the score of the i-th principal component, $X_{\max}$ is the maximum value of the i-th principal component, and $X_{\min}$ is the minimum value of the i-th principal component.

(2) $$W_{i}{=P}_{i}/\overset{n}{\underset{i=1}{\sum }}P_{i}\;\;\;\;\;\;\;\;\;\;i=1,\;2,\cdots \cdots n$$

where $W_{i}$ is the weight of the i-th principal component, and $P_{i}$ is the contribution rate of the i-th principal component.

(3) $$D=\overset{n}{\underset{i=1}{\sum }}[U(X_{i}){\;\times \;W}_{i}]\;\;\;\;\;i=1,\;2,\;\ldots \ldots n$$

where D is the comprehensive evaluation value for regeneration capacity.

## 5. Conclusions and Prospects

This study established complete in vitro regeneration systems for six Chinese large-flowered chrysanthemum cultivars, and optimized genotype-specific technical protocols covering the whole process from explant sterilization to callus induction, adventitious bud differentiation and plantlet rooting. A multi-index comprehensive evaluation system based on PCA and membership function analysis was constructed, which offers a quantitative reference for cultivar screening and protocol optimization, and quantitatively characterizes genotypic differences in overall tissue culture adaptability and regeneration performance. The results confirmed that regeneration capacity and hormone responsiveness present significant genotypic variation across all cultivation stages, with the cultivar ‘Pushuiliubing’ exhibiting the best comprehensive performance among all tested genotypes. These findings provide reliable technical support for large-scale industrial micropropagation, elite germplasm conservation and molecular breeding of large-flowered chrysanthemum. Future studies will explore eco-friendly sterilizing alternatives and dissect the molecular mechanisms underlying these genotypic differences.

## Figures and Tables

**Table 1:** Effects of 6-BA and NAA on the callus induction rate of leaf explants from six Chinese large-flowered chrysanthemum cultivars (%).

6-BA (mg/L)	NAA (mg/L)	Fushanqiujing	Pushuiliubing	Tangyutianjiao	Yingfengpiaoling	Ziqiangda	Zuijin
1	0.10	52.22 ± 0.96 ^g^	95.56 ± 0.96 ^a^	57.78 ± 2.55 ^ghi^	62.22 ± 0.96 ^i^	66.11 ± 0.96 ^f^	63.33 ± 1.67 ^j^
1	0.30	67.78 ± 0.96 ^f^	97.78 ± 0.96 ^a^	69.45 ± 2.55 ^e^	68.89 ± 0.96 ^gh^	100.00 ± 0.00 ^a^	67.22 ± 0.96 ^hi^
1	0.50	68.33 ± 1.67 ^f^	88.89 ± 0.96 ^b^	57.22 ± 0.96 ^hi^	76.11 ± 2.55 ^de^	74.44 ± 0.96 ^c^	90.56 ± 0.96 ^b^
1	0.70	47.78 ± 2.55 ^h^	85.56 ± 3.47 ^c^	55.56 ± 0.96 ^hi^	100.00 ± 0.00 ^a^	62.22 ± 0.96 ^g^	87.78 ± 0.96 ^c^
1	0.90	38.89 ± 2.55 ^i^	82.78 ± 1.92 ^c^	51.67 ± 1.67 ^jk^	66.11 ± 0.96 ^h^	42.22 ± 0.96 ^j^	86.11 ± 0.96 ^cd^
2	0.10	52.78 ± 2.55 ^g^	35.56 ± 0.96 ^h^	50.55 ± 2.55 ^k^	59.45 ± 2.55 ^i^	34.44 ± 0.96 ^k^	76.11 ± 2.55 ^f^
2	0.30	95.00 ± 1.67 ^a^	37.22 ± 0.96 ^h^	87.78 ± 2.55 ^b^	92.22 ± 3.85 ^b^	81.11 ± 0.96 ^b^	81.11 ± 0.96 ^e^
2	0.50	91.11 ± 2.55 ^b^	42.22 ± 0.96 ^g^	91.67 ± 1.67 ^a^	93.89 ± 0.96 ^b^	72.22 ± 0.96 ^d^	98.89 ± 0.96 ^a^
2	0.70	78.33 ± 3.34 ^d^	30.56 ± 1.93 ^i^	61.11 ± 1.92 ^fg^	70.00 ± 2.89 ^fg^	62.78 ± 0.96 ^g^	85.00 ± 1.67 ^d^
2	0.90	53.89 ± 1.92 ^g^	29.44 ± 0.96 ^i^	58.89 ± 2.55 ^gh^	65.56 ± 0.96 ^h^	50.56 ± 0.96 ^i^	71.11 ± 1.92 ^g^
3	0.10	69.44 ± 1.93 ^ef^	32.22 ± 1.92 ^i^	54.44 ± 0.96 ^ij^	72.78 ± 1.92 ^ef^	42.22 ± 0.96 ^j^	68.33 ± 1.67 ^hi^
3	0.30	72.22 ± 3.47 ^e^	40.55 ± 2.55 ^g^	82.78 ± 1.92 ^c^	77.78 ± 0.96 ^d^	52.22 ± 0.96 ^i^	76.11 ± 0.96 ^f^
3	0.50	85.56 ± 0.96 ^c^	52.78 ± 0.96 ^e^	73.89 ± 0.96 ^d^	81.11 ± 2.55 ^c^	68.89 ± 0.96 ^e^	69.44 ± 0.96 ^gh^
3	0.70	91.67 ± 1.67 ^ab^	61.67 ± 1.67 ^d^	72.78 ± 0.96 ^d^	90.56 ± 1.93 ^b^	57.78 ± 1.92 ^h^	66.11 ± 1.92 ^i^
3	0.90	85.00 ± 1.67 ^c^	45.56 ± 1.93 ^f^	62.78 ± 2.55 ^f^	72.78 ± 0.96 ^ef^	51.11 ± 1.92 ^i^	62.22 ± 0.96 ^j^

Note: Values are presented as mean ± standard deviation. Different lowercase letters within the same column indicate significant differences at *p* < 0.05 based on Duncan’s multiple-range test. The same notation applies to all subsequent tables.

**Table 2:** Effects of 6-BA and NAA on the adventitious bud differentiation rate of leaf explants from six Chinese large-flowered chrysanthemum cultivars (%).

6-BA (mg/L)	NAA (mg/L)	Fushanqiujing	Pushuiliubing	Tangyutianjiao	Yingfengpiaoling	Ziqiangda	Zuijin
1	0.10	11.11 ± 0.96 ^e^	33.33 ± 1.67 ^h^	26.11 ± 0.96 ^c^	0.00 ± 0.00 ^h^	14.44 ± 0.96 ^c^	0.00 ± 0.00 ^e^
1	0.30	18.89 ± 0.96 ^b^	71.11 ± 0.96 ^c^	61.66 ± 2.89 ^a^	3.89 ± 0.96 ^g^	13.33 ± 1.67 ^c^	66.67 ± 1.67 ^a^
1	0.50	19.44 ± 1.93 ^b^	17.78 ± 0.96 ^j^	21.66 ± 2.89 ^d^	22.22 ± 1.92 ^d^	5.00 ± 1.67 ^d^	33.89 ± 0.96 ^b^
1	0.70	14.44 ± 0.96 ^c^	12.22 ± 2.55 ^k^	3.89 ± 0.96 ^g^	41.11 ± 2.55 ^b^	3.89 ± 0.96 ^d^	10.56 ± 1.93 ^d^
1	0.90	13.89 ± 0.96 ^cd^	7.22 ± 0.96 ^L^	0.00 ± 0.00 ^h^	47.22 ± 1.92 ^a^	3.33 ± 0.00 ^d^	0.00 ± 0.00 ^e^
2	0.10	3.33 ± 1.67 ^g^	61.11 ± 2.55 ^d^	0.00 ± 0.00 ^h^	0.00 ± 0.00 ^h^	4.44 ± 0.96 ^d^	0.00 ± 0.00 ^e^
2	0.30	7.22 ± 0.96 ^f^	100.00 ± 0.00 ^a^	17.78 ± 2.55 ^e^	0.00 ± 0.00 ^h^	37.22 ± 0.96 ^a^	33.89 ± 0.96 ^b^
2	0.50	12.22 ± 0.96 ^de^	52.22 ± 0.96 ^e^	19.44 ± 0.96 ^de^	6.11 ± 2.55 ^f^	24.45 ± 2.55 ^b^	20.56 ± 0.96 ^c^
2	0.70	12.78 ± 0.96 ^cde^	47.78 ± 0.96 ^f^	0.00 ± 0.00 ^h^	10.56 ± 1.93 ^e^	14.44 ± 0.96 ^c^	0.00 ± 0.00 ^e^
2	0.90	26.11 ± 0.96 ^a^	39.45 ± 2.55 ^g^	0.00 ± 0.00 ^h^	26.11 ± 0.96 ^c^	0.00 ± 0.00 ^e^	0.00 ± 0.00 ^e^
3	0.10	0.00 ± 0.00 ^h^	26.11 ± 0.96 ^i^	0.00 ± 0.00 ^h^	0.00 ± 0.00 ^h^	0.00 ± 0.00 ^e^	0.00 ± 0.00 ^e^
3	0.30	3.89 ± 0.96 ^g^	27.78 ± 2.55 ^i^	41.11 ± 0.96 ^b^	0.00 ± 0.00 ^h^	0.00 ± 0.00 ^e^	32.78 ± 0.96 ^b^
3	0.50	4.44 ± 0.96 ^g^	87.23 ± 2.54 ^b^	7.22 ± 0.96 ^f^	0.00 ± 0.00 ^h^	4.44 ± 0.96 ^d^	0.00 ± 0.00 ^e^
3	0.70	18.33 ± 1.67 ^b^	26.11 ± 0.96 ^i^	0.00 ± 0.00 ^h^	0.00 ± 0.00 ^h^	0.00 ± 0.00 ^e^	0.00 ± 0.00 ^e^
3	0.90	3.89 ± 0.96 ^g^	25.56 ± 0.96 ^i^	0.00 ± 0.00 ^h^	0.00 ± 0.00 ^h^	0.00 ± 0.00 ^e^	0.00 ± 0.00 ^e^

**Table 3:** Effects of 6-BA and NAA on the adventitious bud number per explant of leaf explants from six Chinese large-flowered chrysanthemum cultivars.

6-BA (mg/L)	NAA (mg/L)	Fushanqiujing	Pushuiliubing	Tangyutianjiao	Yingfengpiaoling	Ziqiangda	Zuijin
1	0.10	0.13 ± 0.01 ^e^	1.36 ± 0.12 ^e^	1.07 ± 0.06 ^c^	0.00 ± 0.00 ^f^	0.15 ± 0.00 ^d^	0.00 ± 0.00 ^e^
1	0.30	0.39 ± 0.03 ^c^	0.73 ± 0.03 ^g^	2.52 ± 0.05 ^a^	0.05 ± 0.02 ^ef^	0.14 ± 0.02 ^d^	2.72 ± 0.12 ^a^
1	0.50	0.59 ± 0.06 ^a^	0.21 ± 0.03 ^i^	0.66 ± 0.11 ^d^	0.47 ± 0.03 ^d^	0.06 ± 0.01 ^ef^	1.04 ± 0.04 ^b^
1	0.70	0.46 ± 0.01 ^b^	0.13 ± 0.02 ^i^	0.09 ± 0.03 ^g^	0.91 ± 0.06 ^b^	0.08 ± 0.02 ^e^	0.23 ± 0.04 ^d^
1	0.90	0.30 ± 0.02 ^d^	0.08 ± 0.01 ^i^	0.00 ± 0.00 ^h^	1.92 ± 0.11 ^a^	0.07 ± 0.01 ^ef^	0.00 ± 0.00 ^e^
2	0.10	0.08 ± 0.03 ^ef^	2.41 ± 0.15 ^c^	0.00 ± 0.00 ^h^	0.00 ± 0.00 ^f^	0.05 ± 0.02 ^f^	0.00 ± 0.00 ^e^
2	0.30	0.08 ± 0.02 ^ef^	6.09 ± 0.09 ^a^	0.57 ± 0.06 ^e^	0.00 ± 0.00 ^f^	0.77 ± 0.02 ^a^	0.36 ± 0.03 ^c^
2	0.50	0.27 ± 0.04 ^d^	1.59 ± 0.04 ^d^	0.41 ± 0.03 ^f^	0.07 ± 0.03 ^ef^	0.51 ± 0.04 ^b^	0.41 ± 0.03 ^c^
2	0.70	0.56 ± 0.08 ^a^	0.98 ± 0.03 ^f^	0.00 ± 0.00 ^h^	0.11 ± 0.01 ^e^	0.30 ± 0.02 ^c^	0.00 ± 0.00 ^e^
2	0.90	0.59 ± 0.08 ^a^	0.82 ± 0.07 ^g^	0.00 ± 0.00 ^h^	0.55 ± 0.04 ^c^	0.00 ± 0.00 ^g^	0.00 ± 0.00 ^e^
3	0.10	0.00 ± 0.00 ^g^	0.81 ± 0.05 ^g^	0.00 ± 0.00 ^h^	0.00 ± 0.00 ^f^	0.00 ± 0.00 ^g^	0.00 ± 0.00 ^e^
3	0.30	0.09 ± 0.03 ^ef^	0.57 ± 0.03 ^h^	1.27 ± 0.07 ^b^	0.00 ± 0.00 ^f^	0.00 ± 0.00 ^g^	0.36 ± 0.03 ^c^
3	0.50	0.06 ± 0.03 ^efg^	2.72 ± 0.16 ^b^	0.16 ± 0.01 ^g^	0.00 ± 0.00 ^f^	0.07 ± 0.03 ^ef^	0.00 ± 0.00 ^e^
3	0.70	0.57 ± 0.07 ^a^	1.08 ± 0.08 ^f^	0.00 ± 0.00 ^h^	0.00 ± 0.00 ^f^	0.00 ± 0.00 ^g^	0.00 ± 0.00 ^e^
3	0.90	0.04 ± 0.01 ^fg^	0.79 ± 0.02 ^g^	0.00 ± 0.00 ^h^	0.00 ± 0.00 ^f^	0.00 ± 0.00 ^g^	0.00 ± 0.00 ^e^

**Table 4:** Effects of exogenous IBA or NAA on the rooting of six Chinese large-flowered chrysanthemum cultivars.

Index	IBA (mg/L)	NAA (mg/L)	Fushanqiujing	Pushuiliubing	Tangyutianjiao	Yingfengpiaoling	Ziqiangda	Zuijin
Rooting rate (%)	0	0	97.78 ± 1.92 ^a^	80.00 ± 3.33 ^c^	81.11 ± 1.92 ^b^	100.00 ± 0.00 ^a^	92.22 ± 1.92 ^c^	97.78 ± 1.92 ^a^
	0.1	0	85.56 ± 1.93 ^d^	97.78 ± 1.92 ^a^	57.78 ± 1.92 ^c^	95.56 ± 1.93 ^b^	0.00 ± 0.00 ^d^	91.11 ± 1.92 ^b^
	0.3	0	0.00 ± 0.00 ^f^	90.00 ± 3.33 ^b^	0.00 ± 0.00 ^f^	88.89 ± 1.92 ^c^	0.00 ± 0.00 ^d^	0.00 ± 0.00 ^d^
	0.5	0	0.00 ± 0.00 ^f^	0.00 ± 0.00 ^e^	0.00 ± 0.00 ^f^	80.00 ± 3.33 ^e^	0.00 ± 0.00 ^d^	0.00 ± 0.00 ^d^
	0.7	0	0.00 ± 0.00 ^f^	0.00 ± 0.00 ^e^	0.00 ± 0.00 ^f^	78.89 ± 1.92 ^e^	0.00 ± 0.00 ^d^	0.00 ± 0.00 ^d^
	0	0.1	92.22 ± 1.92 ^b^	88.89 ± 1.92 ^b^	85.56 ± 1.93 ^a^	85.56 ± 1.93 ^d^	93.33 ± 3.34 ^bc^	92.23 ± 3.86 ^b^
	0	0.3	88.89 ± 1.92 ^c^	91.11 ± 1.92 ^b^	46.67 ± 3.34 ^d^	81.11 ± 1.92 ^e^	94.44 ± 1.93 ^bc^	84.45 ± 3.85 ^c^
	0	0.5	86.67 ± 3.34 ^cd^	81.11 ± 1.92 ^c^	44.44 ± 1.93 ^d^	0.00 ± 0.00 ^f^	98.89 ± 1.92 ^a^	81.11 ± 1.92 ^c^
	0	0.7	81.11 ± 1.92 ^e^	75.55 ± 3.85 ^d^	35.56 ± 1.93 ^e^	0.00 ± 0.00 ^f^	95.56 ± 1.93 ^b^	0.00 ± 0.00 ^d^
Mean root number	0	0	7.48 ± 0.15 ^a^	4.69 ± 0.20 ^e^	6.31 ± 0.33 ^b^	7.95 ± 0.07 ^a^	5.17 ± 0.14 ^b^	5.38 ± 0.14 ^a^
	0.1	0	5.20 ± 0.13 ^e^	10.53 ± 0.12 ^a^	3.70 ± 0.19 ^c^	7.26 ± 0.21 ^b^	0.00 ± 0.00 ^c^	3.32 ± 0.15 ^c^
	0.3	0	0.00 ± 0.00 ^g^	6.66 ± 0.28 ^c^	0.00 ± 0.00 ^f^	4.35 ± 0.14 ^c^	0.00 ± 0.00 ^c^	0.00 ± 0.00 ^f^
	0.5	0	0.00 ± 0.00 ^g^	0.00 ± 0.00 ^g^	0.00 ± 0.00 ^f^	3.10 ± 0.19 ^e^	0.00 ± 0.00 ^c^	0.00 ± 0.00 ^f^
	0.7	0	0.00 ± 0.00 ^g^	0.00 ± 0.00 ^g^	0.00 ± 0.00 ^f^	1.73 ± 0.12 ^g^	0.00 ± 0.00 ^c^	0.00 ± 0.00 ^f^
	0	0.1	6.39 ± 0.07 ^b^	6.59 ± 0.15 ^c^	6.68 ± 0.15 ^a^	3.46 ± 0.12 ^d^	5.22 ± 0.20 ^b^	4.32 ± 0.15 ^b^
	0	0.3	6.13 ± 0.17 ^c^	7.66 ± 0.18 ^b^	3.04 ± 0.12 ^d^	2.57 ± 0.18 ^f^	5.24 ± 0.19 ^b^	3.13 ± 0.12 ^d^
	0	0.5	5.47 ± 0.19 ^d^	6.23 ± 0.24 ^d^	2.91 ± 0.16 ^d^	0.00 ± 0.00 ^h^	5.62 ± 0.17 ^a^	1.20 ± 0.03 ^e^
	0	0.7	3.42 ± 0.14 ^f^	4.36 ± 0.22 ^f^	1.82 ± 0.23 ^e^	0.00 ± 0.00 ^h^	5.48 ± 0.22 ^a^	0.00 ± 0.00 ^f^
Mean root length (mm)	0	0	37.42 ± 0.79 ^a^	33.15 ± 0.65 ^e^	23.23 ± 0.41 ^b^	42.23 ± 0.43 ^a^	21.19 ± 0.51 ^d^	48.97 ± 0.30 ^a^
	0.1	0	21.21 ± 0.32 ^e^	57.64 ± 0.34 ^a^	22.24 ± 0.54 ^c^	38.98 ± 0.45 ^b^	0.00 ± 0.00 ^e^	41.19 ± 0.46 ^c^
	0.3	0	0.00 ± 0.00 ^g^	46.16 ± 0.73 ^c^	0.00 ± 0.00 ^g^	23.23 ± 0.66 ^c^	0.00 ± 0.00 ^e^	0.00 ± 0.00 ^f^
	0.5	0	0.00 ± 0.00 ^g^	0.00 ± 0.00 ^g^	0.00 ± 0.00 ^g^	9.07 ± 0.28 ^e^	0.00 ± 0.00 ^e^	0.00 ± 0.00 ^f^
	0.7	0	0.00 ± 0.00 ^g^	0.00 ± 0.00 ^g^	0.00 ± 0.00 ^g^	2.50 ± 0.29 ^g^	0.00 ± 0.00 ^e^	0.00 ± 0.00 ^f^
	0	0.1	27.06 ± 0.54 ^b^	45.57 ± 0.70 ^c^	27.80 ± 0.23 ^a^	14.13 ± 0.53 ^d^	22.32 ± 0.41 ^c^	45.57 ± 0.58 ^b^
	0	0.3	24.92 ± 0.36 ^c^	47.45 ± 0.64 ^b^	11.61 ± 0.37 ^d^	7.25 ± 0.58 ^f^	23.41 ± 0.32 ^b^	26.98 ± 0.39 ^d^
	0	0.5	23.87 ± 0.40 ^d^	36.98 ± 0.33 ^d^	8.17 ± 0.19 ^e^	0.00 ± 0.00 ^h^	24.28 ± 0.38 ^a^	21.85 ± 0.24 ^e^
	0	0.7	3.38 ± 0.14 ^f^	26.67 ± 0.45 ^f^	4.21 ± 0.17 ^f^	0.00 ± 0.00 ^h^	23.72 ± 0.68 ^ab^	0.00 ± 0.00 ^f^

**Table 5:** Eigenvalues and eigenvectors of principal components derived from nine individual in vitro regeneration indices.

	Principal Component Factor		
	1	2	3
Survival rate	0.633	−0.757	−0.127
Contamination rate	−0.422	0.9	0.074
Death rate	−0.483	−0.706	0.131
Callus induction rate	−0.797	0.551	−0.031
Adventitious bud differentiation rate	0.857	0.171	0.478
Adventitious bud number per explant	−0.394	−0.135	0.894
Rooting rate	0.578	0.561	−0.344
Mean root number	0.901	0.04	0.032
Mean root length	0.765	0.518	0.343
Eigenvalues	4.066	2.817	1.305
Contribution rate (%)	45.181	31.295	14.503

**Table 6:** Factor scores, membership function values, and comprehensive evaluation *D*-values of principal components for six Chinese large-flowered chrysanthemum cultivars.

Cultivar	*C*1	*C*2	*C*3	*U*1	*U*2	*U*3	*D*-Value	Rank
Fushanqiujing	−0.107	0.296	−0.383	0.283	0.655	0.413	0.432	3
Pushuiliubing	1.874	0.299	0.717	1.000	0.656	0.818	0.853	1
Tangyutianjiao	−0.677	−1.480	1.209	0.076	0.000	1.000	0.197	6
Yingfengpiaoling	−0.426	0.555	−0.580	0.167	0.751	0.340	0.395	4
Ziqiangda	0.224	−0.890	−1.501	0.403	0.218	0.000	0.275	5
Zuijin	−0.888	1.230	0.537	0.000	1.000	0.752	0.464	2

## Data Availability

The original contributions presented in this study are included in the article. Further inquiries can be directed to the corresponding author.

## Appendix A

**Table A1:** ANOVA of survival rate, contamination rate, and death rate.

Dependent Variable	Source of Variance	Sum of Square	Mean Square	F Value	*p*
Survival rate	Cultivar	6512.534	1302.507	142.516	5.94 × 10^−32^
	Sterilization duration	24,127.526	6031.882	659.987	7.69 × 10^−49^
	Cultivar × Sterilization duration	6217.429	310.871	34.014	1.86 × 10^−25^
Contamination rate	Cultivar	8020.106	1604.021	254.756	4.80× 10^−39^
	Sterilization duration	41,839.948	10,459.987	1661.29	1.10 × 10^−60^
	Cultivar × Sterilization duration	4559.038	227.952	36.204	3.46 × 10^−26^
Death rate	Cultivar	763.792	152.758	72.699	3.64 × 10^−24^
	Sterilization duration	6190.461	1547.615	736.526	3.07 × 10^−50^
	Cultivar × Sterilization duration	932.194	46.61	22.182	1.35× 10^−20^

Survival rate R^2^ = 0.985 (adjusted R^2^ = 0.978); contamination rate R^2^ = 0.993 (adjusted R^2^ = 0.990); death rate R^2^ = 0.984 (adjusted R^2^ = 0.977).

**Table A2:** ANOVA of callus induction rate.

Source of Variance	Sum of Square	Mean Square	F Value	*p*
Cultivar	14,375.890	2875.178	938.002	7.75 × 10^−127^
6-BA	1286.552	643.276	209.863	9.09 × 10^−48^
NAA	14,861.985	3715.496	1212.149	6.13 × 10^−129^
Cultivar × 6-BA	34,077.369	3407.737	1111.745	4.60 × 10^−156^
Cultivar × NAA	4285.032	214.252	69.898	8.25 × 10^−74^
6-BA × NAA	4051.436	506.429	165.218	1.07 × 10^−78^
Cultivar × 6-BA × NAA	13,535.38	338.385	110.395	8.68 × 10^−107^

R^2^ = 0.994 (adjusted R^2^ = 0.991).

**Table A3:** ANOVA of adventitious bud differentiation rate and adventitious bud number per explant.

Dependent Variable	Source of Variance	Sum of Square	Mean Square	F Value	*p*
Adventitious bud differentiation rate	Cultivar	36,822.809	7364.562	4617.324	6.03 × 10^−188^
	6-BA	5206.976	2603.488	1632.297	4.29 × 10^−116^
	NAA	15,426.367	3856.592	2417.949	3.23 × 10^−155^
	Cultivar × 6-BA	13,122.869	1312.287	822.758	1.59 × 10^−144^
	Cultivar × NAA	23,848.417	1192.421	747.606	9.45 × 10^−162^
	6-BA × NAA	1992.853	249.107	156.181	8.89 × 10^−77^
	Cultivar × 6-BA × NAA	21,437.286	535.932	336.011	4.91 × 10^−149^
Adventitious bud number per explant	Cultivar	44.233	8.847	4106.195	2.13 × 10^−183^
	6-BA	4.824	2.412	1119.622	2.78 × 10^−102^
	NAA	15.641	3.910	1814.944	3.04 × 10^−144^
	Cultivar × 6-BA	35.805	3.580	1661.898	1.46 × 10^−171^
	Cultivar × NAA	31.847	1.592	739.090	2.62 × 10^−161^
	6-BA × NAA	5.447	0.681	316.023	1.14 × 10^−101^
	Cultivar × 6-BA × NAA	61.088	1.527	708.862	6.95 × 10^−178^

Adventitious bud differentiation rate R^2^ = 0.998 (adjusted R^2^ = 0.996); adventitious bud number per explant R^2^ = 0.998 (adjusted R^2^ = 0.997).

**Table A4:** ANOVA of rooting rate, mean root number, and mean root length.

Dependent Variable	Source of Variance	Sum of Square	Mean Square	F Value	*p*
Rooting rate	Cultivar	15,566.018	3113.204	810.297	7.08 × 10^−84^
	IBA	91,673.282	22,918.320	5965.125	1.10 × 10^−125^
	NAA	24,241.732	6060.433	1577.395	8.71 × 10^−95^
	Cultivar × IBA	39,767.936	1988.397	517.535	1.22 × 10^−97^
	Cultivar × NAA	31,631.891	1581.595	411.654	2.42 × 10^−92^
	IBA × NAA	0	-	-	-
	Cultivar × IBA × NAA	0	-	-	-
Mean root number	Cultivar	185.566	37.113	1824.91	1.44 × 10^−102^
	IBA	514.704	128.676	6327.173	4.65 × 10^−127^
	NAA	152.348	38.087	1872.787	9.50 × 10^−99^
	Cultivar × IBA	206.44	10.322	507.547	3.46 × 10^−97^
	Cultivar × NAA	141.785	7.089	348.588	1.66 × 10^−88^
	IBA × NAA	0	-	-	-
	Cultivar × IBA × NAA	0	-	-	-
Mean root length	Cultivar	7595.567	1519.113	10,361.603	4.65 × 10^−143^
	IBA	18,194.30748	4548.577	31,025.037	2.89 × 10^−164^
	NAA	6763.59104	1690.898	11,533.314	4.29 × 10^−141^
	Cultivar × IBA	7760.12	388.006	2646.52	1.10 × 10^−135^
	Cultivar × NAA	5597.653	279.883	1909.03	4.81 × 10^−128^
	IBA × NAA	0	-	-	-
	Cultivar × IBA × NAA	0	-	-	-

Rooting rate R^2^ = 0.999 (adjusted R^2^ = 0.998); mean root number R^2^ = 0.998 (adjusted R^2^ = 0.998); mean root length R^2^ = 1.000 (adjusted R^2^ = 0.999).
